# Perturbing nuclear glycosylation in the mouse preimplantation embryo slows down embryonic development

**DOI:** 10.1073/pnas.2410520122

**Published:** 2025-04-09

**Authors:** Sara Formichetti, Joana B. Serrano, Urvashi Chitnavis, Agnieszka Sadowska, Na Liu, Ana Boskovic, Matthieu Boulard

**Affiliations:** ^a^Epigenetics and Neurobiology Unit, European Molecular Biology Laboratory (EMBL) Rome, Monterotondo 00015, Italy; ^b^Combined Faculty of Mathematics, Engineering and Natural Sciences, collaboration for Joint PhD Degree between European Molecular Biology Laboratory and Heidelberg University, Heidelberg 69117, Germany

**Keywords:** O-GlcNAc, embryonic genome activation, preimplantation development, gene expression

## Abstract

The main form of intracellular protein glycosylation (O-GlcNAc) is reversible and has been mapped on thousands of cytoplasmic and nuclear proteins, including RNA polymerase II, transcription factors, and chromatin modifiers. The O-GlcNAc modification is catalyzed by a single enzyme known as O-GlcNAc Transferase, that is required for mammalian early development. Yet, neither the regulatory function of protein O-GlcNAcylation in the embryo nor the embryonic O-GlcNAc proteome have been documented. Here, we devised a strategy to enzymatically remove O-GlcNAc from preimplantation embryonic nuclei, where this modification accumulates coincidently with embryonic genome activation (EGA). Unexpectedly, the depletion of nuclear O-GlcNAc to undetectable levels has no impact on EGA, but dampens the transcriptional upregulation of the translational machinery, and triggers a spindle checkpoint response. These molecular alterations were phenotypically associated with a developmental delay starting from early cleavage stages and persisting after embryo implantation, establishing a link between nuclear glycosylation and the pace of embryonic development.

## Significance

Protein function is modulated by chemical modifications, which include sugars. Unlike highly branched extracellular glycosylations, the monosaccharide O-GlcNAc is reversibly added to thousands of intracellular proteins, participating in many fundamental cellular activities, from glycolysis to cell cycle, transcription, and translation. Thus, O-GlcNAc potentially mediates gene expression responses to environmental changes. The mammalian preimplantation embryo undergoes multiple metabolic changes and requires the enzyme responsible for O-GlcNAc for survival. However, O-GlcNAc’s function in this developmental window is unknown. We show that O-GlcNAc increases in the nucleus of the mouse embryo coincidently with the activation of its genome. Remarkably, removing nuclear O-GlcNAc in the zygote causes a slowdown of embryonic development. However, this is not due to the impairment of embryonic genome activation.

Following fertilization, the embryo relies on the payload of RNA and proteins carried by the oocyte. Then, it undergoes genome-wide reprogramming involving changes to chromatin organization as well as reconfiguration of the transcriptome, with the degradation of maternal transcripts and the activation of the embryonic genome. A less studied concomitant phenomenon is the reconfiguration of the proteome, which includes dynamic posttranslational modifications (PTMs). Among PTMs, intracellular glycosylation is poorly characterized in the early mammalian embryo.

The main form of intracellular glycosylation in animals is O-GlcNAcylation, the reversible addition of the monosaccharide O-linked N-acetylglucosamine (O-GlcNAc) to specific threonine and serine residues. A single pair of enzymes catalyzes the deposition and removal of O-GlcNAc, namely O-GlcNAc transferase (OGT) (1, 2) and O-GlcNAc hydrolase (OGA) (3), both encoded by one single gene in most species, including human and mouse (4). In *Drosophila*, loss-of-function of *Ogt* produces a stereotypical developmental phenotype characterized by the expression of homeotic genes outside their normal boundaries in larvae (5). In the mouse, *Ogt* knockout causes a more severe phenotype whereby the maternal inheritance of a nonfunctional copy of *Ogt* leads to embryonic lethality around the blastocyst stage (6), implying that mammalian OGT and/or O-GlcNAc carry out essential functions either for oocyte development or for preimplantation processes. In addition, OGT plays an essential role in cell proliferation independently of its enzymatic activity (7). *Ogt*’s essential nature has obstructed the understanding of O-GlcNAc function in the mammalian embryo, where its relevance for cellular and developmental processes remains largely unknown.

O-GlcNAc is a highly pleiotropic modification, mapped on thousands of mammalian proteins (8) and involved in many pathways, including translation (9, 10), glucose catabolism (11, 12), and the cell cycle (13). In the nucleus, O-GlcNAc modifies numerous transcription factors, among which the developmentally relevant pluripotency master regulators OCT4 (also known as POU5F1) and SOX2 are noteworthy (14, 15). Because O-GlcNAcylation of OCT4 has been shown to be required for its reprogramming activity in vitro (14), O-GlcNAc might regulate the pluripotency gene network during preimplantation development. Another prominent nuclear target of OGT is the C-terminal domain (CTD) of RNA Polymerase II, where O-GlcNAc was mapped on threonine 4 and serine 5 (16). In spite of its presence on key regulators of gene expression, it remains unclear whether O-GlcNAc plays an instructive function in controlling transcription (17). The early developmental arrest resulting from *Ogt* loss-of-function is consistent with a putative role of O-GlcNAc in the first transcriptional event of the embryo, namely the embryonic genome activation (EGA) (18).

Finally, the donor substrate for O-GlcNAc is UDP-GlcNAc, the end product of the hexosamine biosynthetic pathway, which channels 2 to 3% of intracellular glucose and whose flux is responsive to levels of nutrients such as glucose and glutamine (19, 20). For this reason, O-GlcNAc has been recurrently associated with nutrient sensing and cellular adaptation (11, 12). The early mammalian embryo is a biological paragon of a highly metabolically dynamic system. Glucose import is low until the 8-cell stage and glycolysis almost undetectable during preimplantation development, when the embryo mostly relies on oxidative phosphorylation (21). Accordingly, the expression of enzymes participating in oxidative phosphorylation is upregulated at EGA and gradually increases during the following cleavages (22). At implantation, the decrease in oxygen level together with the need for high proliferation rate and biomass production is accompanied by a metabolic switch which favors glycolysis (22). In light of these findings, early development has the potential to be regulated by a metabolically sensitive PTM such as O-GlcNAc.

These hypotheses on the possible roles of mammalian O-GlcNAc in early development are based on in vitro evidence but have not been tested in vivo. This is largely due to the hurdle posed to classical genetics approaches by the requirement of the *Ogt* maternal allele. In this study, we documented the dynamics of O-GlcNAc across key preimplantation stages, which highlighted its enrichment in the nucleus from the 2-cell stage onward. We then deployed a functional assay based on the enzymatic removal of both embryonic and maternally inherited O-GlcNAc from the nuclei of the preimplantation embryo. Unexpectedly, disturbing nuclear O-GlcNAc homeostasis did not affect EGA, nor the gene networks required for blastocyst formation, but slowed down embryonic development, starting from cleavage stages and persisting after embryo implantation.

## Results

### Uncoupled Dynamics of OGT and O-GlcNAc across Mouse Preimplantation Development.

**Fig. 1.**
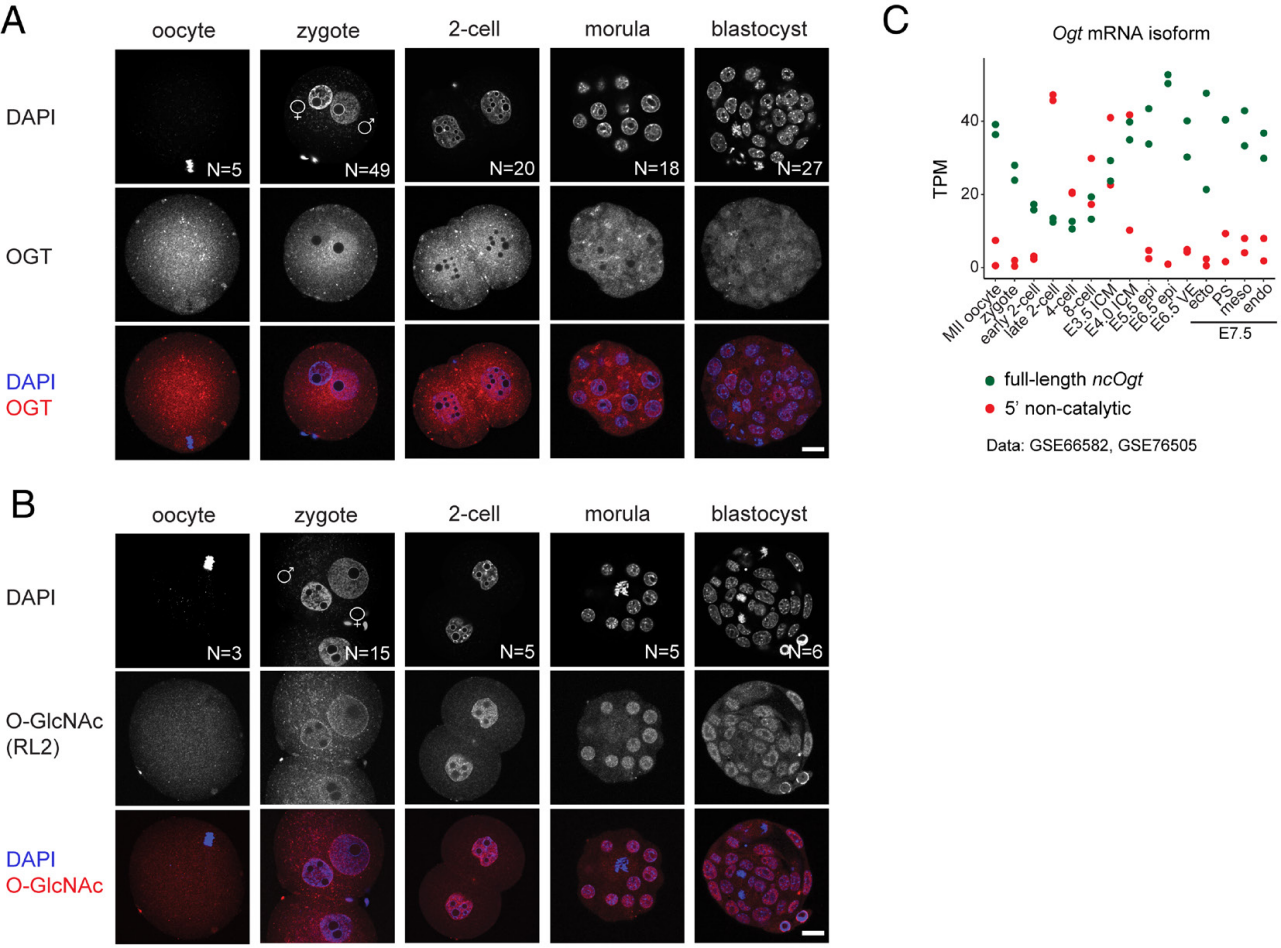
Uncoupled dynamics of OGT and O-GlcNAc across mouse preimplantation development. (*A* and *B*) Immunofluorescence staining of OGT (*A*) and O-GlcNAc (*B*) in MII oocytes, zygotes at various pronuclear stages, 2-cell embryos (22 to 26 h post-IVF), morulae (72 h post-IVF), and blastocysts (96 h post-IVF) generated through IVF. One z-plane is shown for each embryo, except for some zygotes for which two z-planes are shown. Scale bar indicates 20 μm. (*C*) Salmon quantification of the *Ogt* transcript isoforms from mRNA-Seq datasets GSE66582 (31) (MII oocyte to E4.0 ICM) and GSE76505 (32) (E5.5 to E7.5). epi = epiblast, ecto = ectoderm, PS = primitive streak, meso = mesoderm, endo = endoderm.

The dynamics of protein O-GlcNAcylation in the early mouse embryo is unknown. Thus, we set out to determine the spatiotemporal profile of OGT (Fig. 1*A* and *SI Appendix*, Fig. S1*A*), O-GlcNAc (Fig. 1*B* and *SI Appendix*, Fig. S1*B*), and OGA (*SI Appendix*, Fig. S1*C*) across key preimplantation stages. Embryos were generated through in vitro fertilization (IVF) and the following stages were collected for immunofluorescence staining: zygotes at different pronuclear (PN) stages (0 to 4 h post-IVF), late 2-cell stage embryos (22 to 26 h post-IVF), morulae (76 h post-IVF), and late blastocysts (96 h post-IVF). We also collected unfertilized MII oocytes for analysis.

First, we observed an enrichment of the OGT signal in the paternal pronucleus of the zygote compared to the maternal one at all PN stages, consistent with a previous study (25) (Fig. 1*A* and *SI Appendix*, Fig. S1*A*). The same pattern was found with embryos produced by natural mating (*SI Appendix*, Fig. S1*D*). In contrast, the O-GlcNAc signal was equivalently present on both pronuclei (Fig. 1*B* and *SI Appendix*, Fig. S1*B*), as was the signal of the O-GlcNAc hydrolase OGA (*SI Appendix*, Fig. S1*C*), pointing to a possible catalytically independent role of OGT in the zygote. Second, the nuclear-to-cytosolic signal ratio of O-GlcNAc markedly increases from the 2-cell stage onward, a result that was observed with different antibodies (Fig. 1*B* and *SI Appendix*, Fig. S1*E*). The OGT signal was instead roughly equivalent between the nucleus and the cytosol from the 2-cell stage onward (Fig. 1*A*), while OGA was more abundant in the nucleus than in the cytosol from the zygote stage and throughout preimplantation development (*SI Appendix*, Fig. S1*C*). The subnuclear pattern of OGA and O-GlcNAc displayed a noticeable feature: OGA was mutually exclusive with DAPI-dense foci and the same was observed for the foci with the most intense O-GlcNAc signal (*SI Appendix*, Fig. S1*F*). This suggests that nuclear O-GlcNAc-modified proteins and their hydrolase could be excluded from condensed chromatin and/or proteins bound to A/T-rich DNA. Also noteworthy, OGT, OGA, and O-GlcNAc were all undetectable on metaphase chromosomes, while the OGT but not OGA was enriched on the oocyte meiotic spindle (oocytes in Fig. 1 *A* and *B* and *SI Appendix*, Fig. S1 *C* and *E*), as previously reported (26).

In the zygote, O-GlcNAc displayed an intense signal at the nuclear membrane with the RL2 antibody (Fig. 1*B* and *SI Appendix*, Fig. S1*B*). This is likely attributable to the highly O-GlcNAcylated state of the FG nucleoporins subunits of the nuclear pore complex (NPC) (27), since this antibody (anti-O-GlcNAc RL2) was raised against rat NPC-lamina nuclear fraction (28). However, two other antibodies show a wide-spread zygotic pronuclear signal (*SI Appendix*, Fig. S1*E*), and an enrichment at perinucleolar foci in the zygote and in the 2-cell embryo (*SI Appendix*, Fig. S1 *E*, *G*, and *H*). The perinucleolar signal, lost after enzymatic removal of O-GlcNAc (*SI Appendix*, Fig. S1*H*), overlapped with the OGA signal (*SI Appendix*, Fig. S1*G*), suggesting an active remodeling of nucleoli-proximal clusters of O-GlcNAcylated proteins in the zygote.

The monoclonal antibody against OGT used in this study recognizes its C-terminus, thus all reported OGT isoforms, which include the nuclear-cytoplasmic one (ncOGT) in addition to a mitochondrial isoform and a shorter isoform of unknown localization and function (29, 30). To gain insight into OGT isoform usage in the early embryo, we analyzed publicly available mRNA-Seq data spanning mouse pre- and postimplantation stages (31, 32). We noted that the full-length transcript encoding ncOGT is the only one present in the oocyte; after fertilization, its levels gradually diminish and start to rise again only from the 8-cell stage. Instead, reads coverage and an annotation-independent splicing analysis support the production of a shorter form of *Ogt* terminating inside intron 4 of the full-length isoform, from EGA and until the 8-cell stage (Fig. 1*C* and *SI Appendix*, Fig. S1 *I* and *J*). This transcript could correspond to one of the shorter N-terminal isoforms annotated in Ensembl (in red in *SI Appendix*, Fig. S1*I*), which are predicted to be either noncoding or at the very least catalytically inactive due to the absence of the C-terminal catalytic domain. Of note, the retention of intron 4 by the full-length catalytically active isoform was shown to increase with higher O-GlcNAc levels and was associated with nuclear degradation of the transcript, in turn maintaining O-GlcNAc homeostasis (23).

The same analysis for *Oga* showed that there is a small amount of maternal *Oga* transcripts in the zygote. During preimplantation stages, two mRNA isoforms are produced, at relatively low levels: a full-length one and a nonfunctional one [excluding exon 11 and the C-terminal exons of the longest isoform (24)]. A major increase in functional embryonic *Oga* transcripts is observed around implantation (*SI Appendix*, Fig. S1 *K* and *L*).

In summary, the subcellular pattern of the O-GlcNAc modification is highly dynamic during mouse preimplantation development, and distinct from that of OGT. The concentration of the latter appears to be tightly controlled in the early embryo, first by its confinement in the paternal pronucleus and then through alternative production of RNA isoforms, which results in the absence of a catalytically active embryonic OGT before the 8-cell stage. Therefore, the nuclear O-GlcNAc pattern at earlier stages must be established by the maternal OGT and O-GlcNAc payloads and by the differential presence in the various subcellular compartments of the hydrolase OGA.

### Effective Enzymatic Depletion of O-GlcNAc from Mouse Embryonic Nuclei.

The high abundance of O-GlcNAc-modified proteins in the nucleus from the 2-cell stage onward (Fig. 1*B*) prompted us to interrogate the function of O-GlcNAcylation specifically on nuclear proteins during preimplantation development.

**Fig. 2.**
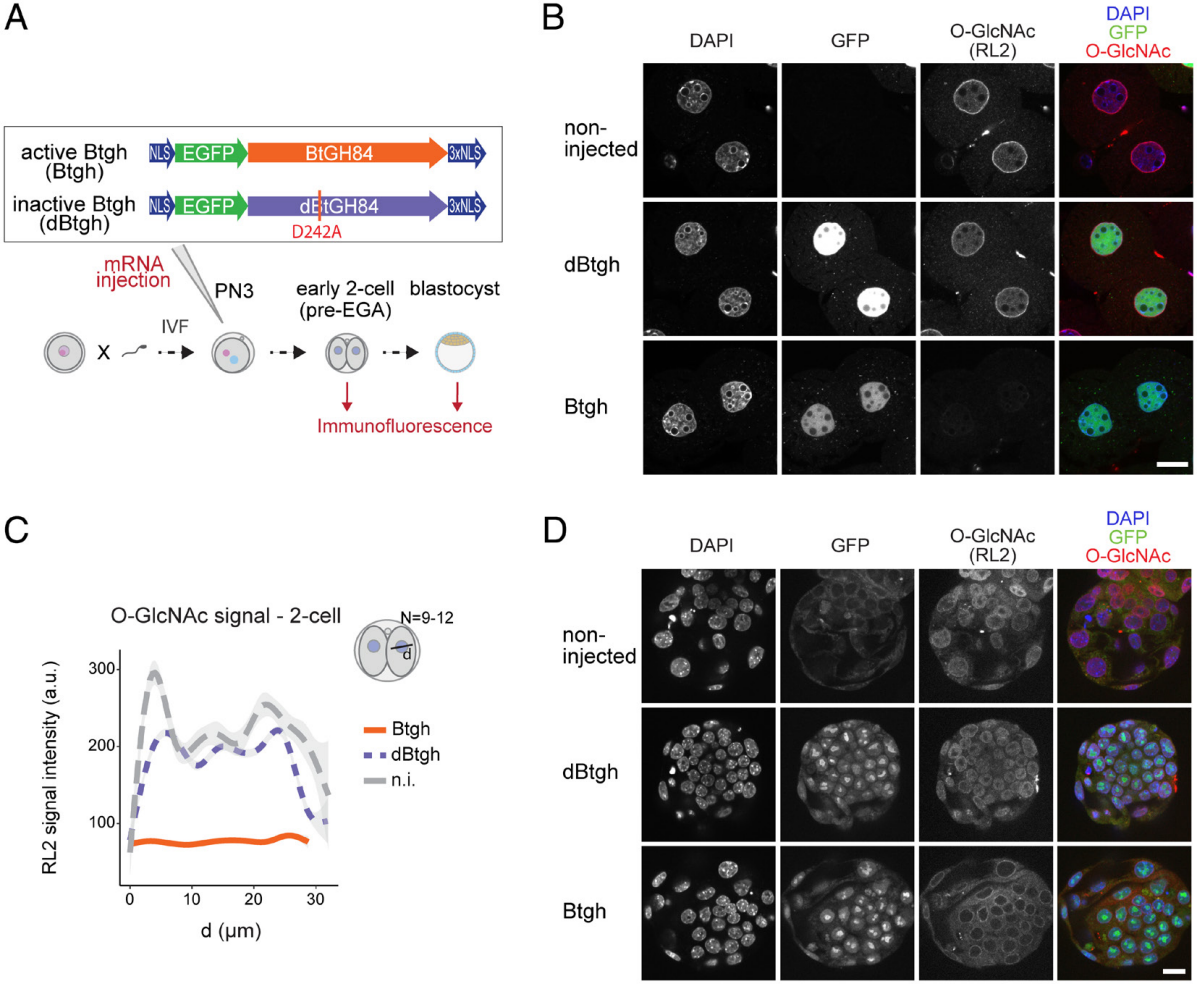
Effective enzymatic depletion of O-GlcNAc from mouse embryonic nuclei. (*A*) Experimental design for results in (*B*–*D*). Zygotes were generated through IVF and injected 2 h later with the mRNA of the active or inactive form of NLS-EGFP-BtGH84-3xNLS (Btgh and dBtgh, respectively), or left uninjected. The three groups of embryos were cultured ex vivo and fixed either as pre-EGA 2-cell or at the blastocyst stage (96 h post-IVF). (*B* and *C*) Confocal imaging of pre-EGA 2-cell embryos (*B*) and blastocysts (*D*) from zygotes injected with Btgh/dBtgh or noninjected, stained with an anti-O-GlcNAc antibody (RL2). One z-plane is shown for each embryo. Scale bar indicates 20 μm. The GFP signal comes from the Btgh/dBtgh protein fused to GFP. (*C*) Quantification of the O-GlcNAc signal across single nuclei of 2-cell embryos from the experiment in (*B*). N = 9-12 nuclei per experimental group were quantified, from two independent microinjection experiments.

Toward this goal, we needed an experimental system that overcomes the embryonic lethality caused by maternal *Ogt* loss-of-function and, importantly, that eliminates the payload of protein O-GlcNAcylation inherited from the oocyte. Our strategy was to enzymatically remove the O-GlcNAc moieties from the zygote onward by expressing a recombinant bacterial O-GlcNAcase targeted to the nucleus. To this end, we leveraged the homologous O-GlcNAcase from the human gut symbiont *Bacteroides thetaiotaomicron* (BtGH84), whose structure and catalytic mechanism have been well characterized in vitro (33). Because *B. thetaiotaomicron* lacks OGT’s mammalian orthologs, BtGH84 is highly unlikely to interfere with the function of the endogenous OGT and OGA or to bind their endogenous interactors. We fused BtGH84 to nuclear localization signals on both extremities and to an EGFP reporter at the N terminus to monitor the expression at the single-cell level. We injected the mRNA of this construct (henceforth called “Btgh”) into zygotes 2 h after IVF (~PN3; Fig. 2*A*). Two other groups of embryos were used to control for potential side effects due to the injection procedure and the translation of an exogenous protein: noninjected embryos and embryos injected with a catalytically inactive Btgh construct, bearing a single amino acid mutation (D242A) lowering both V_max_ and affinity to the substrate (K_m_) to negligible values (henceforth “dBtgh” for “dead Btgh”) (33). After injection of Btgh, the O-GlcNAc signal was dramatically reduced to nearly undetectable levels already at the early 2-cell stage (i.e., before EGA; Fig. 2 *B* and *C*), while it remained comparable to noninjected embryos following dBtgh expression. We confirmed the pervasive O-GlcNAc depletion using different antibodies against O-GlcNAc (clone HGAC85 and mix of rabbit monoclonal antibodies; *SI Appendix*, Fig. S1 *H* and *M* respectively). The depletion of nuclear O-GlcNAc upon Btgh injection was still substantial at the morula stage (*SI Appendix*, Fig. S1*M*) and, importantly, it persisted until the late blastocyst stage (embryonic day 4; E4), the last stage assessed with immunostaining (Fig. 2*D*).

### Nuclear O-GlcNAc Depletion Does Not Affect Differentiation but Slows Down Development.

Having validated the efficiency of our perturbation method, we investigated the effect of nuclear O-GlcNAc depletion on the progression of preimplantation development. We found no impact on blastocyst formation rate ex vivo at E4 (*SI Appendix*, Fig. S2*A*), nor on the specification of the trophectoderm (TE) and inner cell mass (ICM) lineages (*SI Appendix*, Fig. S2*B*). Hence, physiological levels of nuclear O-GlcNAc are dispensable for progression to the blastocyst stage. This result was unexpected because glycosylation of the master regulator of pluripotency OCT4 was shown to be required for its reprogramming function in vitro (14).

**Fig. 3.**
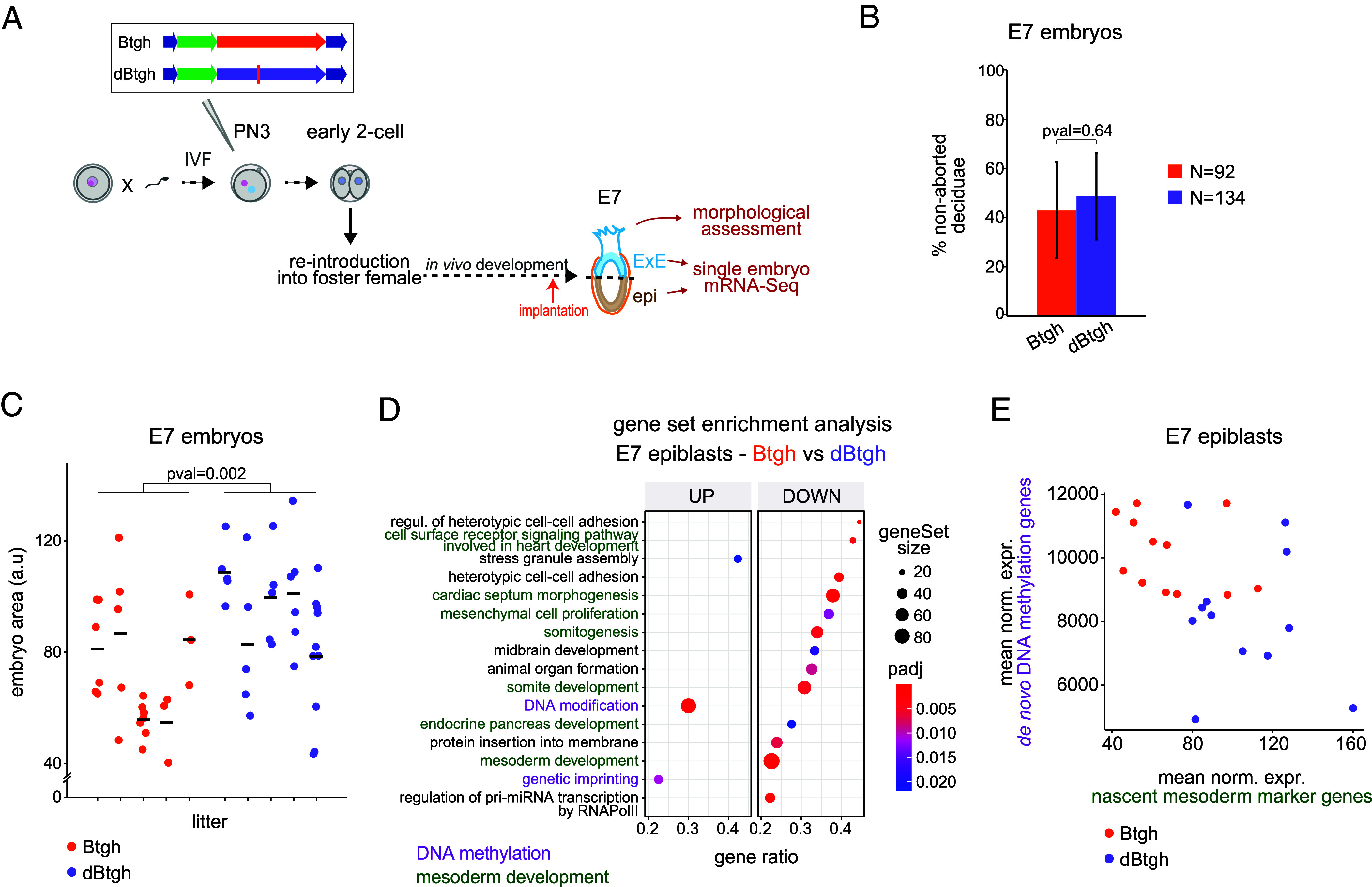
Nuclear O-GlcNAc depletion does not affect differentiation but slows down development. (*A*) Experimental design for postimplantation results. Zygotes were generated through IVF and injected 2 h later with the mRNA of either Btgh or dBtgh, then cultured ex vivo overnight and the next morning transferred to pseudopregnant females. Six days later the embryos were dissected, imaged, and cut into two halves, largely corresponding to the epi and ExE tissues, respectively. The two halves were separately processed for single-embryo mRNA-Seq. (*B*) Percentage of Btgh/dBtgh-injected embryos recovered at E7. N = 92 and 134 total injected 2-cell embryos transferred to pseudopregnant females for Btgh and dBtgh, respectively. Bar heights indicate the average, and error bars indicate the SD of five replicates of the microinjection experiment. *P*-value was computed using unpaired Student’s *t* test, assuming unequal variance. (*C*) Size of the Btgh/dBtgh-injected E7 embryos for all dissected litters, as measured by the area of the embryos in the images taken with the stereomicroscope (*SI Appendix*, Fig. S2 *C*, *Upper* part). Each column shows a litter. *P*-value was computed using the unpaired Wilcoxon rank sum exact test. (*D*) GSEA of gene expression changes in E7 epiblasts derived from Btgh-injected embryos vs. dBtgh-injected controls. Among the significant BP gene ontology (GO) terms, the 16 with the highest Normalized Enrichment Score are shown, ordered by gene ratio and colored based on manual grouping of cellular functions. Size of dots is proportional to the number of total genes of a GO term. Gene ratio = fraction of total genes of the GO term which are concordantly changing between the two conditions. (*E*) Mean DESeq2-normalized counts of E7 mesoderm markers from ref. 34 vs. mean DESeq2-normalized counts of enzymes associated with de novo DNA methylation (*Dnmt3a*, *Dnmt3b*, *Tet1*) in single E7 epiblasts.

We set out to test whether nuclear O-GlcNAc perturbation affects pre- and peri-implantation development in physiological conditions of nutrients and gas. To this end, zygotes injected with either the Btgh or dBtgh mRNA were grown ex vivo overnight, and the resulting early 2-cell stage embryos were then surgically transferred into pseudopregnant females and dissected 6 d later (at embryonic day 7; Fig. 3*A*). The percentage of embryos recovered at E7 between the two groups of injections was not significantly different (Btgh: 43%, N = 92, dBtgh: 48%, N = 134; *P*-value = 0.64; Fig. 3*B*), indicating that the depletion of nuclear O-GlcNAc had no detrimental impact on implantation.

Although staging of the E7 embryos based on the appearance of the mesoderm (35) showed a similar percentage of embryos to be Pre-, Early-, or Mid-Streak stages in both groups (*SI Appendix*, Table S1), nuclear O-GlcNAc-depleted embryos were on average smaller (*P* = 0.02, unpaired Wilcoxon rank sum exact test; Fig. 3*C*). This difference was mainly driven by 2 out of 5 litters of Btgh-injected embryos; thus the phenotype might be partially penetrant. To further assess this morphological observation with more sensitive molecular data, we microdissected individual E7 conceptuses from the two experimental groups into the epiblast (epi) and extraembryonic ectoderm (ExE) halves, and processed the single embryonic halves for mRNA-Seq (*SI Appendix*, Fig. S2*C*). We interpret gene expression differences between the two groups of postimplantation embryos as a consequence of the depletion of O-GlcNAc during preceding stages rather than as a result of active ongoing removal because the injected Btgh/dBtgh mRNA was no longer detectable at E7, while a low reads count could still be detected in E4 blastocyst data (*SI Appendix*, Fig. S2*D*). Moreover, a compensatory change in expression of the O-GlcNAc enzymes has been reported whenever O-GlcNAc homeostasis is disturbed (23, 36); because *Ogt* was upregulated and *Oga* downregulated in Btgh-injected embryos at the morula and blastocyst stages, while this phenomenon disappeared at E7 (*SI Appendix*, Fig. S2*E*), our data suggest that O-GlcNAc homeostasis has been restored after implantation.

While no difference in gene expression was observed in the ExE between the two groups, several genes were differentially expressed in the epi (adj. *P*-value < 0.05, any log_2_FC; differentially expressed genes (DEGs); *SI Appendix*, Fig. S2*F*). Gene set enrichment analysis (GSEA) uncovered downregulation of genes associated with mesoderm development and upregulation of genes involved in DNA methylation upon Btgh injection (Fig. 3*D*). The development of the mesoderm characterizes the transition between E6.5 and E7.5 (34). On the other hand, the onset of mammalian gastrulation coincides with de novo DNA methylation, with the enzymes involved in this process peaking in expression at E5.5 and then gradually decreasing (*SI Appendix*, Fig. S2*G*). Hence, the transcriptome of the epiblasts from Btgh-injected E7 embryos resembles that of a slightly earlier developmental stage, implying a developmental delay. In agreement, all significantly upregulated genes are found to decrease in expression during the transition from E5.5 to E7.5 in unperturbed embryos, while the opposite was true for the downregulated DEGs (*SI Appendix*, Fig. S2*H*). We used the expression of E7 mesodermal markers (34) and DNA methylation enzymes (*Dnmt3a*/*b*, *Tet1*) in single-embryos to assess the penetrance of the developmental delay suggested by both morphological assessment and differential gene expression analysis. Nine out of twelve epiblasts isolated from Btgh-injected embryos showed lower expression of E7 mesodermal markers compared to controls (and in most cases also higher expression of *Dnmt*s and *Tet1*; Fig. 3*E*), thus supporting a highly penetrant developmental delay after implantation upon preimplantation nuclear O-GlcNAc depletion.

### Nuclear O-GlcNAc Is Dispensable for EGA.

**Fig. 4.**
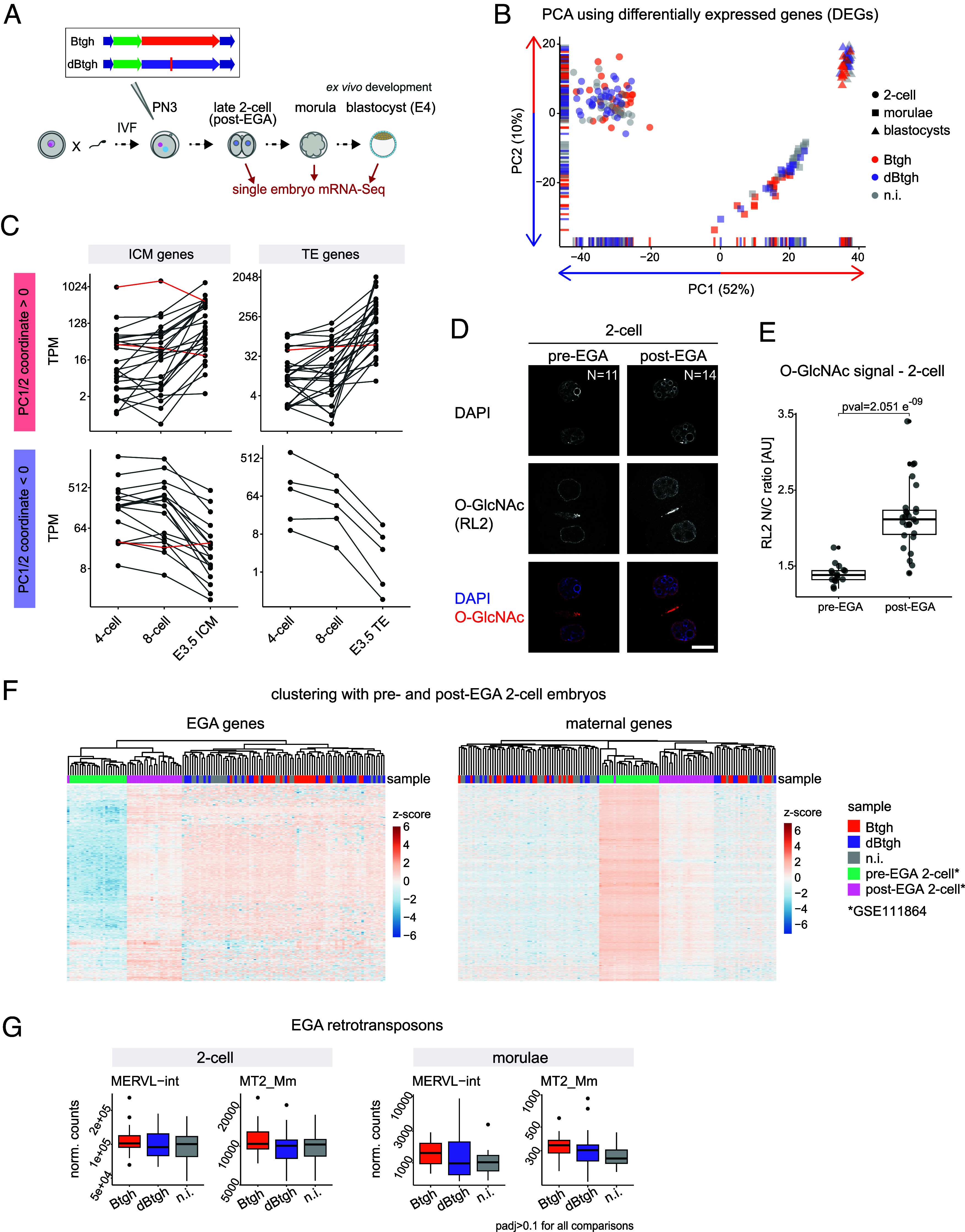
Nuclear O-GlcNAc is dispensable for EGA. (*A*) Experimental design for preimplantation results in Figs. 4 and 5. Zygotes were generated through IVF and injected 2 h later with the mRNA of Btgh or dBtgh or left uninjected. The three groups of embryos were cultured ex vivo until the post-EGA 2-cell, morula, and blastocyst stages and collected for single embryo mRNA-Seq. (*B*) PCA of single embryo transcriptomes at the indicated stages, using all DEGs in O-GlcNAc-depleted embryos (adj. *P*-value < 0.05 in the Btgh vs. dBtgh or Btgh vs. noninjected comparison at any stage). The variance explained by each principal component is indicated in parentheses. N = 85 two-cell embryos, 56 morulae, and 48 blastocysts. (*C*) TPM across cleavage stages [mRNA-Seq data from GSE66582 (31) and GSE76505 (32)] of the 50 and 30 genes mostly contributing to, respectively, the variance explained by PC1 and PC2. Genes were divided based on their positive (*Upper* row) or negative (*Bottom* row) coordinates on the respective PC, and plotted in two groups based on the E3.5 blastocyst tissue where they showed higher TPM (ICM: inner cell mass; TE: trophectoderm). The only three genes whose expression goes in the opposite direction to the rest are plotted in red. (*D*) Immunofluorescence staining of O-GlcNAc (RL2 antibody) in pre- and post-EGA 2-cell embryos generated through IVF. One z-plane is shown for each embryo. Scale bar indicates 20 μm. The total number of embryos imaged per stage is indicated. (*E*) Quantification of the nuclear to cytosolic (N/C) ratio of O-GlcNAc signal in single blastomeres of the 2-cell embryos shown in (*D*). Data are from two replicates of the IVF experiment, which were pooled in one single plot. *P*-value was computed using the unpaired Wilcoxon rank sum exact test. (*F*) Heatmap of the three experimental groups of single 2-cell embryos from this study, together with pre-EGA and post-EGA 2-cell embryos generated through ICSI (GSE111864) (37). All samples were pooled in one DESeq2 dataset, the data normalized using DESeq2 and log_2_ transformed. Then, all strictly EGA genes (*Left*) and all strictly maternal genes (*Right*) with mean of DESeq2-normalized counts ≥10 in the combined 2-cell dataset (this study plus GSE111864) were used to build the heatmap. Heatmap values are scaled by rows and both genes and samples are clustered based on Pearson correlation. (*G*) DESeq2-normalized counts of two types of retrotransposons associated with mouse EGA. MERVL-int denotes the full-length MERVL element, MT2_Mm the MERVL 5′ LTR. Padj = adj. *P*-value computed using the DESeq2 Wald test and corrected for multiple testing using the Benjamini and Hochberg method. Y-axes ticks are in log_10_ scale.

The postimplantation developmental delay upon zygotic Btgh injection should be a consequence of a perturbation caused by the absence of nuclear O-GlcNAc during preceding stages (*SI Appendix*, Fig. S2 *D* and *E*). Hence, we analyzed individual O-GlcNAc-depleted preimplantation embryos collected at the post-EGA 2-cell, morula, and blastocyst stages via single-embryo mRNA-Seq (Fig. 4*A*). Notably, principal component analysis (PCA) of embryonic transcriptomes from the three stages revealed that O-GlcNAc-depleted morulae were shifted farther away from the blastocysts compared to their control counterparts (Fig. 4*B*). The direction of PC1 and PC2 correlated with developmental progression, as supported by the dynamics of genes contributing to these PCs (Fig. 4*C*). Therefore, this result points to a developmental delay already captured in the morula transcriptomes. We conclude that nuclear O-GlcNAc removal across preimplantation stages does not prevent cell differentiation nor implantation, but causes a slight developmental delay that starts during embryonic cleavages and is still measurable postimplantation.

We observed that the nuclear O-GlcNAc signal in the preimplantation embryo sharply increases at the 2-cell stage (Fig. 1*B* and *SI Appendix*, Fig. S1*E*). This does not happen at the time of syngamy but later during the 2-cell stage, coincidently with EGA (Fig. 4 *D* and *E*). This observation, together with the modification of RNA PolII CTD by O-GlcNAc (16), prompted us to investigate whether nuclear O-GlcNAc depletion alters the process of EGA. To this end, we clustered the 2-cell transcriptomes from our dataset with published mRNA-Seq data of single 2-cell stage embryos collected 18 h (pre-EGA) and 28 h (post-EGA) after intracytoplasmic sperm injection (ICSI) (37). We defined “strictly EGA” genes by intersecting published EGA genes (38) and genes significantly upregulated (adj. *P*-value < 0.05 and log_2_FC > 1) from 18 h to 28 h; we defined “strictly maternal” genes as the ones significantly downregulated (adj. *P*-value < 0.05 and log_2_FC < −1) from 18 h to 28 h. Notably, both the activation of embryonic genes and the degradation of maternal transcripts clustered the samples from our experiments with post-EGA 2-cell embryos, with no additional clustering due to O-GlcNAc removal (Fig. 4*F* and *SI Appendix*, Fig. S3*A*). Therefore, the transcriptional maternal-to-zygotic transition is overall unaffected by nuclear O-GlcNAc depletion. In good agreement, the expression of known EGA markers (*Sp110*, *Zfp352*, *Zscan4c*, *B020004J07Rik*, *Arg2*, *Tcstv3*) was either unchanged or increased upon O-GlcNAc depletion (*SI Appendix*, Fig. S3*B*).

A spike in the expression of the MERVL class of LTR retrotransposons is one of the defining features of the 2-cell stage transcriptome (39). Both full-length MERVL (MERVL-int) and MT2 5′ LTRs (MT2_Mm) are associated with EGA (40, 41). The two types of MERVL transcripts were equally expressed or slightly upregulated between Btgh-injected and control embryos (Fig. 4*G*). Furthermore, we found no differentially expressed retrotransposons in O-GlcNAc-depleted 2-cell embryos (*SI Appendix*, Fig. S3*C*). O-GlcNAc-depleted morulae showed a few significantly deregulated retrotransposons (adj. *P*-value < 0.1; *SI Appendix*, Fig. S3*C*), but the difference in expression was very low in magnitude (0.1 < log_2_FC < 0.4). Most of the retrotransposons deregulated in morulae are dynamically expressed across preimplantation stages (*SI Appendix*, Fig. S3*D*), thus their transcript level in O-GlcNAc-depleted embryos could derive from the developmental delay. The trend of sustained upregulation of MERVL-int and MT2-Mm elements up to the morula stage after depletion of O-GlcNAc complies with this interpretation (Fig. 4*G*). As another example, the significantly upregulated MTA_Mm-int is an ERVL-MaLR element typical of the 1-cell embryo (42) which should be downregulated between the 4-cell and 8-cell stage (*SI Appendix*, Fig. S3*D*).

Taken together, our results demonstrate that both EGA and the degradation of maternal transcripts are globally unaffected by nuclear O-GlcNAc removal. Hence, the developmental delay observed in the morula transcriptome starts after the completion of the maternal-to-embryonic transition.

### Misregulation of Mitotic and Translation-Related Genes in Nuclear O-GlcNAc-Depleted Embryos.

**Fig. 5.**
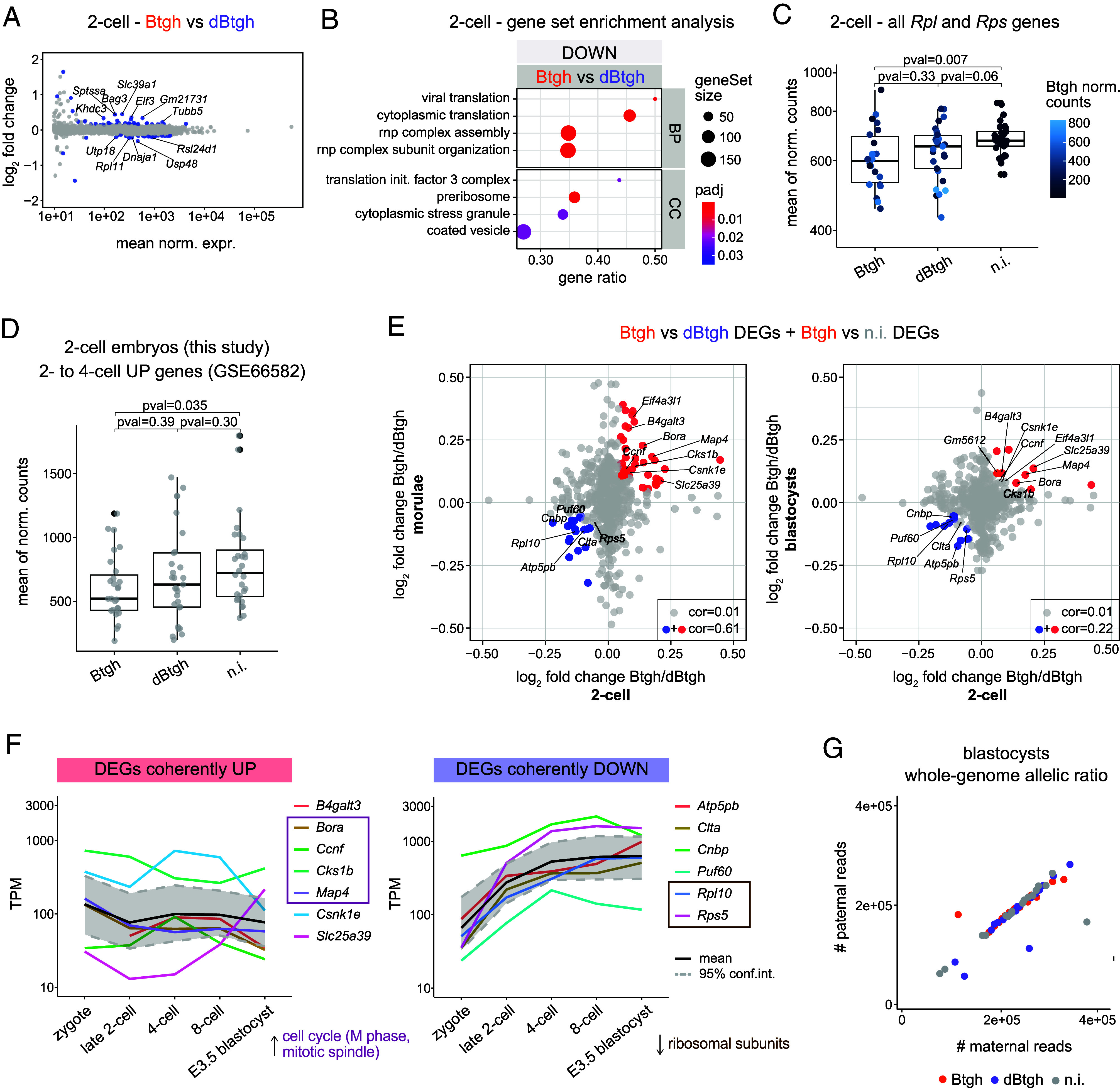
Misregulation of mitotic and translation-related genes in nuclear O-GlcNAc-depleted embryos. (*A*) MA-plots from DESeq2 differential gene expression in 2-cell embryos injected by Btgh vs. dBtgh. Only genes with mean of DESeq2-normalized counts ≥ 10 are shown. All genes with adj. *P*-value < 0.05, any log_2_FC are colored. Genes standing out are labeled. (*B*) GSEA of gene expression changes in nuclear O-GlcNAc-depleted 2-cell embryos injected by Btgh vs. dBtgh. The eight significant GO terms with the highest Normalized Enrichment Score are shown for BP and Cellular Component (CC) GOs, ordered by gene ratio. The size of dots is proportional to the number of total genes of a GO term. Gene ratio = fraction of total genes of the GO term which are concordantly changing between the two conditions. (*C*) Single-embryo mean of DESeq2-normalized counts of all *Mus musculus* ribosomal protein genes from the Ribosomal Protein Gene Database (43). Y-axis ticks are in log_2_ scale. Dots are colored based on DESeq2-normalized counts of the Btgh/dBtgh RNAs. (*D*) Single-embryo mean of DESeq2-normalized counts of genes which are strongly upregulated between the post-EGA 2-cell and the 4-cell stage in publicly available mRNA-Seq data (GSE66582) (31). One outlier noninjected embryo is excluded from both plot and statistical test. (*C* and *D*) *P*-value was computed using the Wilcoxon rank sum exact test. (*E*) Comparison of transcriptional deregulation across preimplantation stages, shown as a scatter plot of the log_2_ fold changes (between Btgh-injected and dBtgh-injected embryos) measured at each stage for all DEGs in O-GlcNAc-depleted embryos (adj. *P*-value < 0.05 in the Btgh vs. dBtgh or Btgh vs. noninjected comparison at any stage; DEGs with DESeq2-normalized counts <10 at any stage were excluded from the plot). DEGs with abs(log_2_FC) > 0.05 at both stages and abs(log_2_FC) ≥ 0.1 in at least one of the two stages are colored (coherently deregulated between two stages); among those, the ones with abs(log_2_FC) > 0.05 at all stages are labeled (coherently deregulated at all stages). cor = Pearson correlation. (*F*) Expression dynamics of DEGs coherently deregulated at all stages [labeled in (*E*); the two pseudogenes were excluded] throughout preimplantation development [mRNA-Seq data from GSE66582 (31) and GSE76505 (32)]. The two biological replicates per stage were averaged. The mean for all genes is drawn, as well as the 95% CI, computed using basic nonparametric bootstrap (R function “mean.cl.boot”). The Y-axis shows TPM and ticks are in log_10_ scale. (*G*) Parental contribution to the whole autosomal genome in single Btgh/dBtgh-injected and noninjected FVB/PWD F1 blastocysts, measured as the total number of maternal (FVB) and paternal (PWD) mRNA-Seq reads. Outliers from the diagonal display allelic imbalance, thus represent potential aneuploid embryos.

Although the depletion of nuclear O-GlcNAc did not measurably impact EGA, post-EGA 2-cell stage embryos displayed a widespread change in gene expression of low magnitude (Fig. 5*A* and *SI Appendix*, Fig S4*A*). GSEA did not identify any specific upregulated pathway, while downregulated genes tend to be involved in the process of translation (Fig. 5*B*). Accordingly, genes encoding ribosomal proteins (*Rpl12*, *Rpl11*, *Rps5*, *Rps15a*, *Rps26*, *Rps2*, *Rpl10*) as well as factors of translation initiation and mRNA metabolism (*Eif4b*, *Eif3a*, *Ythdf2*, *Larp4*) were significantly downregulated after O-GlcNAc depletion (*SI Appendix*, Fig. S4 *B* and *C*). When averaging the expression of all ribosomal protein genes (43), Btgh-injected embryos showed a mild but significant downregulation (*P*-value = 0.007; Fig. 5*C*). The embryonic translational machinery is robustly transcriptionally activated during preimplantation development, particularly at the 2-cell stage (44), as shown by the coordinated increase in the expression of ribosomal protein genes (*SI Appendix*, Fig. S4*D*). Thus, a subtle developmental delay already starting in post-EGA 2-cell embryos upon O-GlcNAc depletion could potentially explain the downregulation of translation-related genes. We tested this hypothesis by plotting the expression of the genes with a higher than twofold increase between the 2- and 4-cell stages (excluding those directly involved in translation). O-GlcNAc-depleted 2-cell embryos showed a lower average expression of these genes (*P*-value = 0.035; Fig. 5*D*) albeit this did not correlate with the lower expression of ribosomal protein genes in single embryos (*SI Appendix*, Fig. S4*E*). Therefore, we cannot establish whether the lower mRNA level of translation-associated gene is a consequence or a cause of a developmental delay already starting at the late 2-cell stage.

To assess the impact of nuclear O-GlcNAc depletion on the global rate of protein synthesis, we quantified the incorporation of a puromycin analog (O-propargyl-puromycin, OPP) at the 2-cell stage and at the morula stage after Btgh injection vs. control groups. There were no significant differences in OPP signal between the three experimental groups of embryos neither at the 2-cell stage nor in the morulae, where a decrease in OPP incorporation was observed in the Btgh-vs. dBtgh-injected groups for only one replicate (*SI Appendix*, Fig. S4 *F*–*J*). This result indicates that the mild transcriptional downregulation of translation-associated genes does not measurably impact global translational rate during the analyzed time window.

At later preimplantation stages, the magnitude of gene expression changes due to O-GlcNAc depletion remained overall small and the highest number of DEGs was found in the morulae (*SI Appendix*, Fig. S4*K*). Notably, when we compared the GSEA of morulae and blastocysts, we found several biological processes (BPs) affected in both stages (*SI Appendix*, Fig. S4*L*). Downregulated genes participate in various essential cellular functions, including the mitochondrial respiratory complex, intracellular and membrane transport, the actomyosin cytoskeleton, the proteasome, and DNA compaction. On the other hand, upregulated genes in morulae and blastocysts were mostly related to the metaphase-to-anaphase cell cycle transition (*SI Appendix*, Fig. S4*L*). Concerning ribosome-related GOs found to be downregulated in de-GlcNAcylated 2-cell embryos (Fig. 5*B*), the direction of their changes in de-GlcNAcylated morulae and blastocysts is the mirror opposite of their changes at corresponding stages in unperturbed embryos from publicly available data (*SI Appendix*, Fig. S4*M*), hence providing further evidence of a developmental delay caused by O-GlcNAc nuclear depletion. Besides ribosome-related genes, we expect that a consistent fraction of the transcriptional differences observed at these later preimplantation stages to be a consequence of the developmental delay upon O-GlcNAc depletion, and we further assess this hypothesis below.

We analyzed the dynamics of DEGs (adj. *P*-value < 0.05 in Btgh-injected embryos vs. either of the two controls at any stage) across the three preimplantation stages. DEGs were poorly overlapping (*SI Appendix*, Fig. S4*N*) and their log_2_FC was uncorrelated between stages (Fig. 5*E*), as expected from three developmentally distant time points with stage-specific transcriptomes. However, a set of DEGs was coherently deregulated from the 2-cell embryo to the morula, and a smaller subset even up to the blastocyst (Fig. 5*E*). Most of the coherently upregulated genes (7 in total) are involved in the transition to the mitotic phase of the cell cycle (*Ccnf*, *Bora*, *Cks1b*) or in the mitotic spindle (*Bora*, *Map4*), but they also include the mitochondrial glutathione transporter *Slc25a39* (45), *B4galt3*, one of the 7 beta-1,4-galactosyltransferases responsible for the synthesis of complex-type N-linked oligosaccharides (46), and Casein Kinase 1 Epsilon (*Csnk1e*), a central component of the circadian clock (47). The persistently downregulated genes (6 in total) include two ribosomal proteins (*Rpl10* and *Rpl5*), Poly(U) Binding Splicing Factor 60 (*Puf60*), involved in pre-mRNA splicing (48), a subunit of mitochondrial ATP synthase (*Atp5pb*), Clathrin Light Chain A (*Clta*), which coats intracellular vesicles, and CCHC-Type Zinc Finger Nucleic Acid Binding Protein (*Cnbp*), involved in transcriptional repression by binding the sterol regulatory element (SRE) (49). The analysis of the RNA level of coherently deregulated genes across preimplantation stages in unperturbed embryos showed that the downregulated ones are normally gradually upregulated throughout preimplantation development, up to 10-fold (Fig. 5 *F*, *Bottom*). Hence, their persistent transcriptional downregulation could be due to their modulation by O-GlcNAc, but could also be contributed by the developmental delay caused by O-GlcNAc depletion, in the case of such a delay already starting in post-EGA 2-cell stage embryos (Fig. 5*D*). These two scenarios are not mutually exclusive.

On the other hand, coherently upregulated genes showed more complex expression dynamics in unperturbed preimplantation embryos (Fig. 5 *F*, *Upper*), thus their misregulation is unlikely a consequence of a delayed transcriptome but rather an acute response to the absence of O-GlcNAc. The upregulation of mitotic genes could indicate a dysfunction in chromosome segregation, leading to aneuploidy. To test this possibility, we quantified the allelic ratio of autosomal genes in single blastocysts depleted of nuclear O-GlcNAc. This was achieved by using F1 hybrid embryos from evolutionary distant parental mouse strains (female FVB/NCrl × male PWD/Ph; *SI Appendix*, Fig. S4*O*). This analysis did not reveal a higher incidence of maternal/paternal allelic imbalance after nuclear O-GlcNAc depletion (Fig. 5*G*).

In summary, the data revealed that nuclear O-GlcNAc depletion from the late zygote causes a developmental delay that starts after the completion of the maternal-to-embryonic transition. From around this time, the transcript level of genes involved in translation starts to be reduced when O-GlcNAc homeostasis is perturbed, without significant consequence on global protein translation rate. This may be a consequence of the developmental delay already starting in the late 2-cell embryo. In addition, spindle checkpoint genes are upregulated throughout preimplantation stages, although this is not associated with major consequences on mitosis.

## Discussion

We established the dynamics and the subcellular localization of O-GlcNAc along with its writer and eraser enzymes across key mouse preimplantation developmental stages. We found that the subcellular localization patterns of OGT and O-GlcNAc are uncoupled. In the zygote, OGT is enriched in the paternal pronucleus compared to the maternal one. Because the payload of zygotic OGT proteins comes from the oocyte, the localization to the paternal pronucleus implies an active process, which could potentially have a functional significance. Of the handful of factors for which such an asymmetrical pattern has been reported, the modification of the DNA by 5-hydroxymethylcytosine (5hmC) is noticeable. 5hmC that is catalyzed by ten-eleven translocation (TET) enzymes is enriched at the paternal pronucleus similarly to OGT and was proposed to contribute to paternal DNA demethylation in the zygote (50–52). TETs are among the most stable OGT interactors (53). The possibility of OGT function in the paternal pronucleus being related to zygotic DNA demethylation is intriguing, perhaps representing a missing player in this incompletely understood process (54). Recently, the conditional genetic deletion of *Ogt* in mouse embryonic stem cells was shown to cause a genome-wide mild reduction of cytosine methylation coupled with an increase in 5hmC (55). Alternatively, OGT interaction with the chromatin of the paternal pronucleus might be relevant for excluding the enzyme from other cellular compartments as a way to regulate O-GlcNAc concentration. It is important to bear in mind that embryonic cleavages are not accompanied by cell growth, thus O-GlcNAc homeostasis during this period might require lowering OGT effective concentration. This hypothesis is consistent with our finding of a low abundance of full-length enzymatically active *Ogt* transcript at EGA, which indicates that, either through alternative splicing or other posttranscriptional mechanisms, the embryonic genome initially produces very little of the catalytically competent *ncOgt* mRNA.

The remarkable increase of nuclear O-GlcNAcylated proteins during the 2-cell stage guided our functional approach, consisting of the injection in the zygote of a nuclear-targeted bacterial homologue of OGA, namely Btgh. This method has three key features that are essential for addressing the function of the O-GlcNAc PTM itself in early mammalian development: First, it does not interfere with OGT function, a critical feature considering the nonenzymatic role of OGT in cell division (7); second, it does not affect oocyte maturation, nor fertilization, an effect observed upon OGA inhibition (26); third, targeting Btgh to the nucleus reduces the risk of inducing cell lethality or proliferation arrest, which until now have obfuscated the role of O-GlcNAc in mammalian embryonic gene expression. Indeed, the importance of mammalian O-GlcNAc in fundamental cellular processes such as the cell cycle has been described (13) and could explain, at least in part, the lethality observed in any proliferating cell type where murine *Ogt* was knocked-out (6, 56) while the deletion of *Ogt* in postmitotic neurons did not affect cell viability (57).

The reduction of nuclear O-GlcNAc to undetectable levels did not interfere with the activation of the embryonic genome, nor with the gene networks responsible for the first embryonic lineage specification. This result was unanticipated because O-GlcNAc is widely believed to contribute to pluripotency and developmental gene expression (14, 58).

Differential gene expression across four developmental stages revealed that removal of nuclear O-GlcNAc reduces the pace of embryonic development, starting from cleavage stages and persisting after implantation. Notably, at the transcriptional level this was associated with the dampening post-EGA of the 2-cell-specific activation of a set of genes whose regulation is closely linked with growth demand, namely translation-related genes, including many ribosomal protein (RP) genes. Cells dedicate a large fraction of their transcription to ribosome biogenesis, hence the need to adapt this effort to nutrient and energy conditions. In eukaryotic cells, rRNA and RP gene transcription is regulated by nutrient sensors such as target of rapamycin (TOR) signaling (59). At the same time, a coordinated ribosomal gene production is necessary to ensure proper growth. Although our experiments cannot establish the directionality of the interplay between the transcriptional downregulation of translation and the observed developmental retardation, the two phenomena are interconnected. We can speculate about possible nuclear O-GlcNAc targets acting on both growth and ribosomal genes, for example factors of the mTORC signaling cascade itself. In the cytosol, an activator of mTORC, named Raptor, has recently been reported to signal glucose status via its O-GlcNAcylation (60). Finally, a hyperactivation of mTORC and mitochondrial function, which could in turn cause the transcriptional downregulation of translation and mitochondrial genes, was described multiple days after *Ogt* KO in mESCs (61). However, the transcriptome of O-GlcNAc-depleted embryos did not show a signature of oxidative stress response. It is also noteworthy that the inhibition of OGT during in vitro neuronal differentiation accelerates the differentiation process, indicating that in a different cellular context, O-GlcNAc could have the opposite effect on developmental pace (62).

We also identified a set of genes linked to the mitotic phase of the cell cycle that is upregulated at all stages after O-GlcNAc removal. Notably, our profiling revealed that OGT is enriched on the oocyte meiotic spindle (Fig. 1*A*). Moreover, a crosstalk between O-GlcNAcylation and phosphorylation has been reported for several proteins associated with mitotic progression (63). It is worth noting that the breakdown of the nuclear envelope at mitosis results in Btgh-free diffusion to the cytosolic compartment during this phase; thus it cannot be excluded that Btgh activity could cause a slight delay in cell division, uncoupled from major mitotic defects, as suggested by the apparently normal cleavage and absence of aneuploidy.

Finally, we wish to underline the challenge of establishing a direct link between O-GlcNAc perturbation and the observed transcriptional and developmental phenotypes. This challenge is due to the pleiotropy of O-GlcNAc, which has the ability to orchestrate a cellular response by acting simultaneously on many targets. The global nuclear removal of O-GlcNAc uncovered the modulation of the pace of embryonic growth as its most phenotypically relevant function in the embryo. Taken together with the associated transcriptional response affecting translation and the cell cycle, this O-GlcNAc function could have a long-reaching significance, since both these processes have been proposed as the molecular basis of species-specific developmental pace (64–66). This study should pave the way for further investigations on this understudied connection between nuclear glycosylation and embryonic growth under homeostatic and challenging conditions.

## Materials and Methods

### In Vitro Fertilization.

In vitro fertilization was performed based on the published protocol (67), with minor modifications, and it is detailed in *SI Appendix*. Four hours after sperm addition to cumulus-oocyte-complexes, zygotes were cleaned from the surrounding cumulus cells and sperm by 5 to 6 washes in KSOM, then cultured in KSOM (Sigma-Aldrich #MR-106-D) in a standard mammalian cell incubator (37 °C, 5% CO_2_) for 2 h prior to microinjections or for 0 to 4 h prior to fixation for immunostaining.

### Zygote Microinjection.

Two hours after the end of IVF, zygotes were transferred to an injection plate with M2 medium (Sigma-Aldrich #M7167) at 37 °C, and subjected to microinjections. Zygotes were microinjected with either pRN3P-NLS-EGFP-Btgh-3xNLS or pRN3P-NLS-EGFP-deadBtgh-3xNLS mRNA, both at 100 ng/µL (for the 2-cell RNA-Seq and IF experiment) or 300 ng/µL (for all other experiments). RNA injections were carried out using a Femtojet microinjector (Eppendorf) at 150 hPa pressure for 0.1 s, with 10 hPa compensation pressure (estimated microinjection volume of 10 pL). After the microinjections, zygotes were washed 3 to 4 times in KSOM and placed back into culture. In the case of Btgh/dBtgh injections, GFP fluorescence was verified the next day and GFP-positive (injected) 2-cell embryos were sorted from the noninjected ones and used for downstream imaging and developmental or transcriptomics analyses.

### Immunofluorescence Staining of Preimplantation Embryos.

MII oocytes (20 h post-hCG), zygotes (0, 2, and 4 h post-IVF, where 0 h is the end of the IVF), 2-cell embryos (pre-EGA: 19 h post-IVF, post-EGA: 28 h post-IVF), morulae (72 h post-IVF), and blastocysts (96 h post-IVF) were fixed in 4% PFA and processed for immunostaining as in ref. 68, detailed in *SI Appendix*. After IF, embryos were immediately mounted on coverslips in Vectashield or, for *SI Appendix*, Fig. S2*B*, in drops of 75% Vectashield in PBS, to preserve the 3D structure. We used Vectashield containing DAPI for staining of the DNA (Vector Laboratories #H-1200).

The primary antibodies used were anti-OGT (abcam #ab177941), anti-OGA (ThermoFisher #PA5-67426), anti-O-GlcNAc clone RL2 (abcam #ab2739 and Merck Millipore #MABS157), anti-O-GlcNAc clone HGAC85 (ThermoFisher #HGAC85), O-GlcNAc MultiMab monoclonal antibodies mix (CST #82332), anti-CDX2 (abcam #ab76541). Dilution of all primary antibodies was 1:200. Secondary antibodies used were A647-conjugated goat anti-rabbit IgG (#A-21244), A488-conjugated goat anti-rabbit IgG (#A-11008), A488-conjugated goat anti-mouse IgG (#A-11001), A555-conjugated goat anti-rabbit IgG (#A-27039), A555-conjugated goat anti-mouse IgG (#A-21424) (all from ThermoFisher). Dilution of all secondary antibodies was 1:500. Fixed immunostained samples were imaged using a Nikon AX scanning confocal (using galvanometric mirrors) or, for *SI Appendix*, Fig. S2*B*, using an X-light V3 Spinning disk confocal. In all images, the DNA signal comes from DAPI. The total number of embryos imaged per stage or experimental group of a specific stage is indicated in each figure, and it comes from at least two independent IVF experiments, except for *SI Appendix*, Fig. S1*D* for which it comes from one replicate.

### Preimplantation Embryo Collection and Library Preparation for Single Embryo Smart-Seq.

Single post-EGA 2-cell embryos (28 h post-IVF), morulae (72 h post-IVF), and blastocysts (96 h post-IVF) were collected in 5 μL of 1× TCL lysis buffer (Qiagen #1031586) containing 1% (v/v) 2-mercaptoethanol (Gibco #31350010) and 0.5 U/µL of SUPERaseIn RNase Inhibitor (ThermoFisher #AM2694). RNA was separated from the surrounding material using RNAClean XP beads (Beckman Coulter #A63987) according to the manufacturer’s protocol for a 96-well plate and small-volume reactions. After the last ethanol wash, the RNA was eluted from RNA beads in 8 µL of H_2_O, 3 µL of which were transferred to a new plate containing 1 µL of dNTPs (BiotechRabbit #BR0600204, 10 mM each) and 1 µL of 10 µM oligo-dT primer (5′–AAGCAGTGGTATCAACGCAGAGTACT30VN–3′). A modified Smart-Seq2 protocol (69) using SuperScript IV RT and tagmentation procedure was used to prepare single-embryo full-length cDNA sequencing libraries (70) (detailed in *SI Appendix*). The pool of cleaned-up cDNA was sequenced in one run (40 bp paired-end mode) on the Illumina NextSeq 500 sequencer. See *SI Appendix*, Table S3 for the number of pooled embryos and average number of reads obtained per embryo at each stage.

### Surgical Transfer of Embryos for In Utero Development, E7 Dissection, and Preparation for Smart-Seq.

CD1 females 7 to 8 wk of age in estrus were mated to vasectomized males, and the day of the vaginal plug was considered to be 0.5 day of pseudopregnancy. Microinjected zygotes were kept in culture overnight. The morning after (the day of vaginal plug), 15 to 20 embryos from each experimental group were surgically transferred into the oviduct of each pseudopregnant female. Six days later (7 d after IVF, for which reason we consider the embryos to be embryonic day 7), the foster mother was killed, and the embryos removed from their implantation sites and dissected in PBS with 10% FBS (PAN/Biotech). Embryos were cleaned by removing Reichardt’s membrane and surrounding maternal cells using 0.01 mm Tungsten needles (F.S.T. #10130-10), then imaged with transillumination on a Leica M205C dissection microscope. For Smart-Seq, embryos were bisected with a glass needle along the epi, ExE boundary, then the two halves collected in 200 µL of 1× RNA/DNA protection buffer (NEB #T2010S) in PBS, snap frozen, and stored at −70 °C until RNA extraction. RNA from single embryonic halves was extracted using the Monarch Total RNA Miniprep Kit (NEB #T2010S), following the manufacturer’s instructions for mammalian whole blood with some modifications: 5 µL of Proteinase K were added to each thawed sample, followed by incubation at room temperature for 15 min. RNA was eluted with 25 µL of 1× TE buffer. For a balanced sequencing design, sex genotyping was performed using cDNA made from 2 µL of each sample. cDNA was synthesized with 100 U of Maxima H Minus Reverse Transcriptase (ThermoFisher #EP0752), using random hexamers and following the manufacturer’s instructions. Sex determination was achieved by cDNA PCR genotyping using three pairs of primers, against the mRNA of *Xist*, *Ddx3y,* and *Eif2s3y* genes, respectively (see *SI Appendix*, Table S4 for primer sequences). The quality of the RNA was checked on an RNA High Sensitivity ScreenTape with TapeStation 4150 (Agilent) and only good-quality samples were used for the Smart-Seq2 library preparation described above and sequencing on a NextSeq 500 (40 bp paired-end mode).

### Analysis of Microscopy Images (Excluding OPP Incorporation Assay).

All steps were performed using ImageJ (71) unless specified. For the quantification of O-GlcNAc signal in 2-cell embryos, the z-plane with the largest nuclear area and highest intensity of DAPI signal was selected for each blastomere nucleus. Then, for Fig. 2*C*: i. a measuring line was drawn across each nucleus and the RL2 signal intensity recorded; ii. all single-nucleus measurements were used to generate a line plot in R using function “geom_smooth” with “gam” method. For Fig. 4*E*: i. an ellipse was drawn inside the cytosol and nucleus of each blastomere and the mean RL2 signal intensity recorded for both compartments; ii. a custom script in R was used to compute the N/C intensity ratios.

The size of E7 embryos was measured from microscopy images as follows. A line was drawn following the contour of each embryo (including epiblast and trophectoderm, excluding the ectoplacental cone; as shown in *SI Appendix*, Fig. S2 *C*, *Upper* part), and the area delimited by the line was recorded. One litter from dBtgh-injected embryos which was a clear outlier (all mid-streak or later stages) was excluded from the resulting plot (Fig. 3*C*).

### Single Embryo Smart-Seq Data Analysis for Single-Copy Genes.

Except for allele-specific analysis, gene counts were obtained using a standard pipeline, detailed together with the differential expression analysis in *SI Appendix*, and available at https://github.com/boulardlab/OGlcNAc_early_embryo_Formichetti2023.

### Analysis of Publicly Available mRNA-Seq Data.

For the analysis of expression levels from datasets GSE66582 (31) and GSE76505 (32), a standard pipeline based on reads alignment to the transcriptome was used, detailed in *SI Appendix* and available at https://github.com/boulardlab/OGlcNAc_early_embryo_Formichetti2023.

For the inspection of reads coverage and the analysis of isoform usage and alternative splicing, fastq files from more runs of the same sample were concatenated before trimming, then mapping was performed using STAR v2.7.8a (72) in single-sample 2-pass mode for higher accuracy, after genome indexing optimized to read length (--sjdbOverhang set to 100). Bigwig files were obtained with deeptools v3.0.2, normalizing to bins per million. For *Ogt* (Fig. 1*C*), Transcriptsper million (TPM) of isoforms ENSMUST00000119299 and ENSMUST00000044475 were averaged (full-length *ncOgt*), as well as TPM of ENSMUST00000153979, ENSMUST00000150161, ENSMUST00000155792, and ENSMUST00000155713 (5′ noncatalytic); ENSMUST00000147635 was excluded from the plot because of no evidence of its presence based on a manual inspection of the Integrative Genomics Viewer (IGV) tracks. For *Oga* (*SI Appendix*, Fig. S1*L*), the isoform containing all exons (ENSMUST00000026243) was considered the full-length isoform; TPM of isoforms ENSMUST00000235508 and ENSMUST00000236960 were averaged (shorter no exon 11); ENSMUST00000235865, ENSMUST00000235448, and ENSMUST00000235936 were excluded from the plot because of no evidence of their presence based on a manual inspection of the IGV tracks.

## Supplementary Material

Appendix 01 (PDF)

## Data Availability

The datasets generated in this study are available at Biostudies under the accession E-MTAB-12981 (73), together with the table of gene counts (featureCounts output) for all samples that passed the filtering steps (last column of *SI Appendix*, Table S3). The previously published data used in this study are available under the accession numbers: GSE76505 (74), GSE66582 (75), and GSE111864 (76). The source code of all bioinformatic analyses performed is available via GitHub at https://github.com/boulardlab/OGlcNAc_early_embryo_Formichetti2023 (77). The plasmids generated in this study are available at www.addgene.org under accession numbers 194469 (78) and 194470 (79). All other data are included in the manuscript and/or *SI Appendix*.
