# Supplementary material for: Perturbing nuclear glycosylation in the mouse preimplantation embryo slows down embryonic development
Source: Proc Natl Acad Sci U S A. 2025 Apr 9;122(15):e2410520122. doi: 10.1073/pnas.2410520122 (PMC12012502; doi:10.1073/pnas.2410520122)
Supplement: Supplementary file 1 — Appendix 01 (PDF) [file pnas.2410520122.sapp.pdf]

PNAS

Supporting Information for
Perturbing nuclear glycosylation in the mouse preimplantation embryo slows down embryonic development

Sara Formichetti[1,2], Joana B. Serrano[1,3], Urvashi Chitnavis[1,3], Agnieszka Sadowska[1], Na Liu[1], Ana Boskovic[1]*, Matthieu Boulard[1,]*

[1] Epigenetics and Neurobiology Unit, European Molecular Biology Laboratory (EMBL), Monterotondo, Italy

[2] Collaboration for joint PhD degree between EMBL and Heidelberg University, Germany

[3] J.B.S. and U.C. contributed equally to this work.

* To whom correspondence may be addressed: Ana Boskovic, Matthieu Boulard

Email: ana.boskovic@embl.it (A.B.), matthieu.boulard@embl.it (M.B.)

This PDF file includes:

- Figures S1 to S4
- Tables S1 to S4
- Supplemental Methods
- Supplemental References

Figure S1. Uncoupled dynamics of OGT and O-GlcNAc across mouse preimplantation development (pages 2-5).

Figure S2. Nuclear O-GlcNAc depletion does not affect differentiation but slows down development (pages 6-7).

Figure S3. Expression of retrotransposons in nuclear O-GlcNAc-depleted and unperturbed preimplantation embryos (pages 8-9).

Figure S4. Misregulation of mitotic and translation-related genes in nuclear O-GlcNAc-depleted embryos (pages 10-13).

Table S1. Staging of E7 embryos based on widefield microscopy images (page 14).

Table S2. Primers and gBlock used for cloning the pRN3P-NLS-EGFP-Btgh/dBtgh-3xNLS plasmids (page 15).

Table S3. Details on the generation and filtering steps of the single embryo Smart-Seq datasets. (page 16).

Table S4. Primers used for PCR genotyping of E7 Btgh/dBtgh-injected embryos' cDNA (page 17)

Supplemental Methods (pages 18-23)

Supplemental References (pages 24-25)

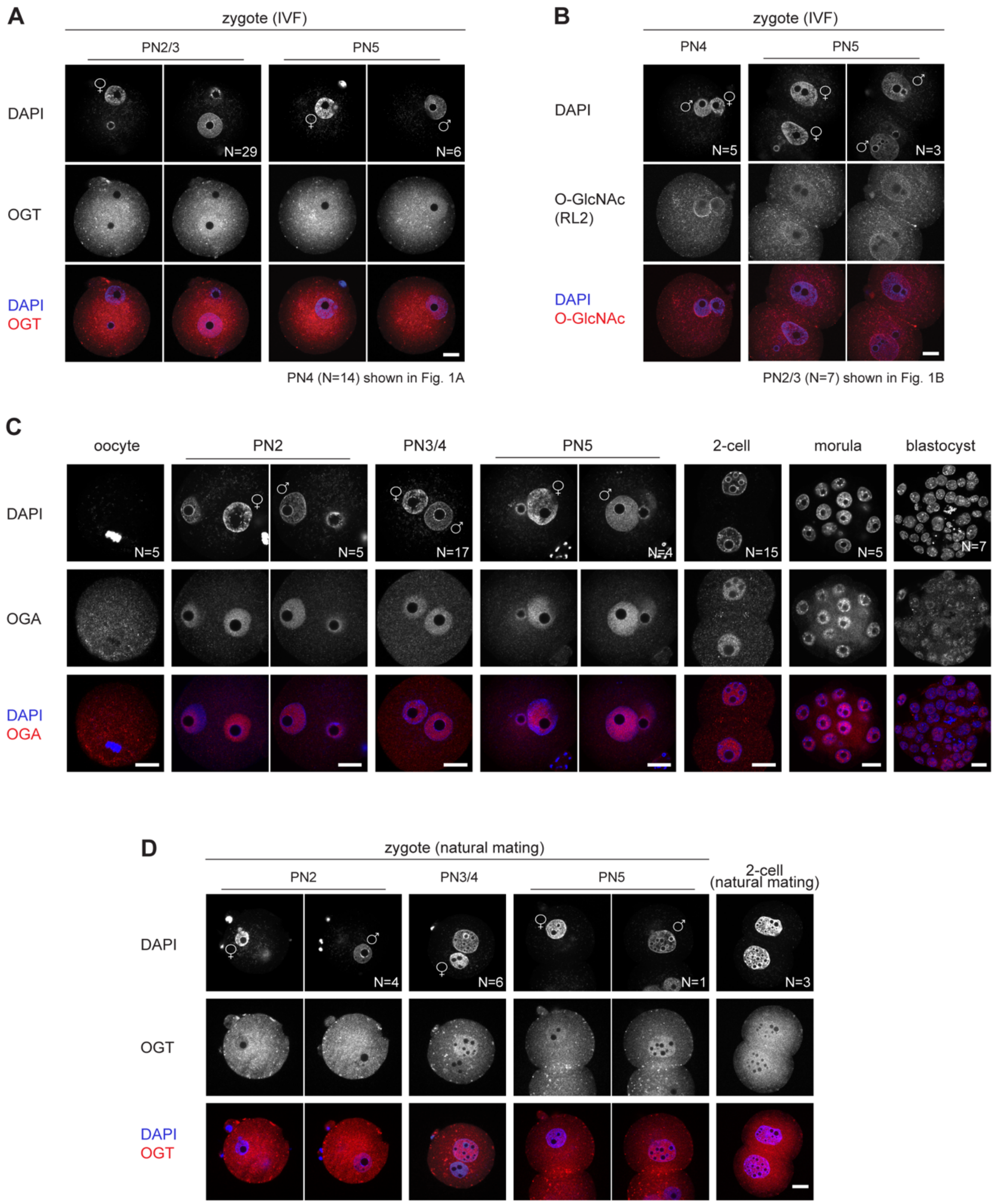

Fig. S1. Uncoupled dynamics of OGT and O-GlcNAc across mouse preimplantation development. *(continued on pages 3-5)*

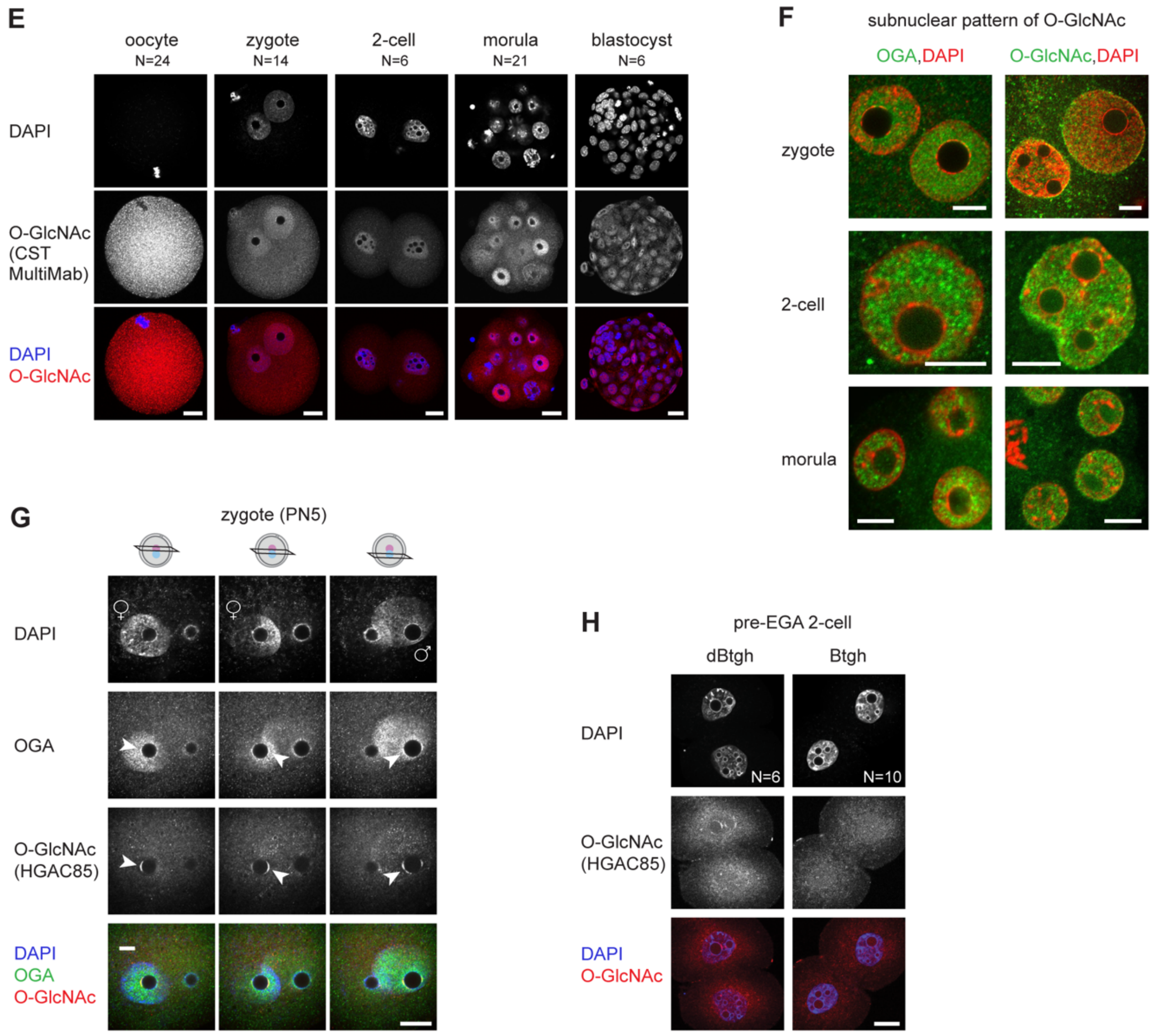

Fig. S1. Uncoupled dynamics of OGT and O-GlcNAc across mouse preimplantation development. *(continued)*

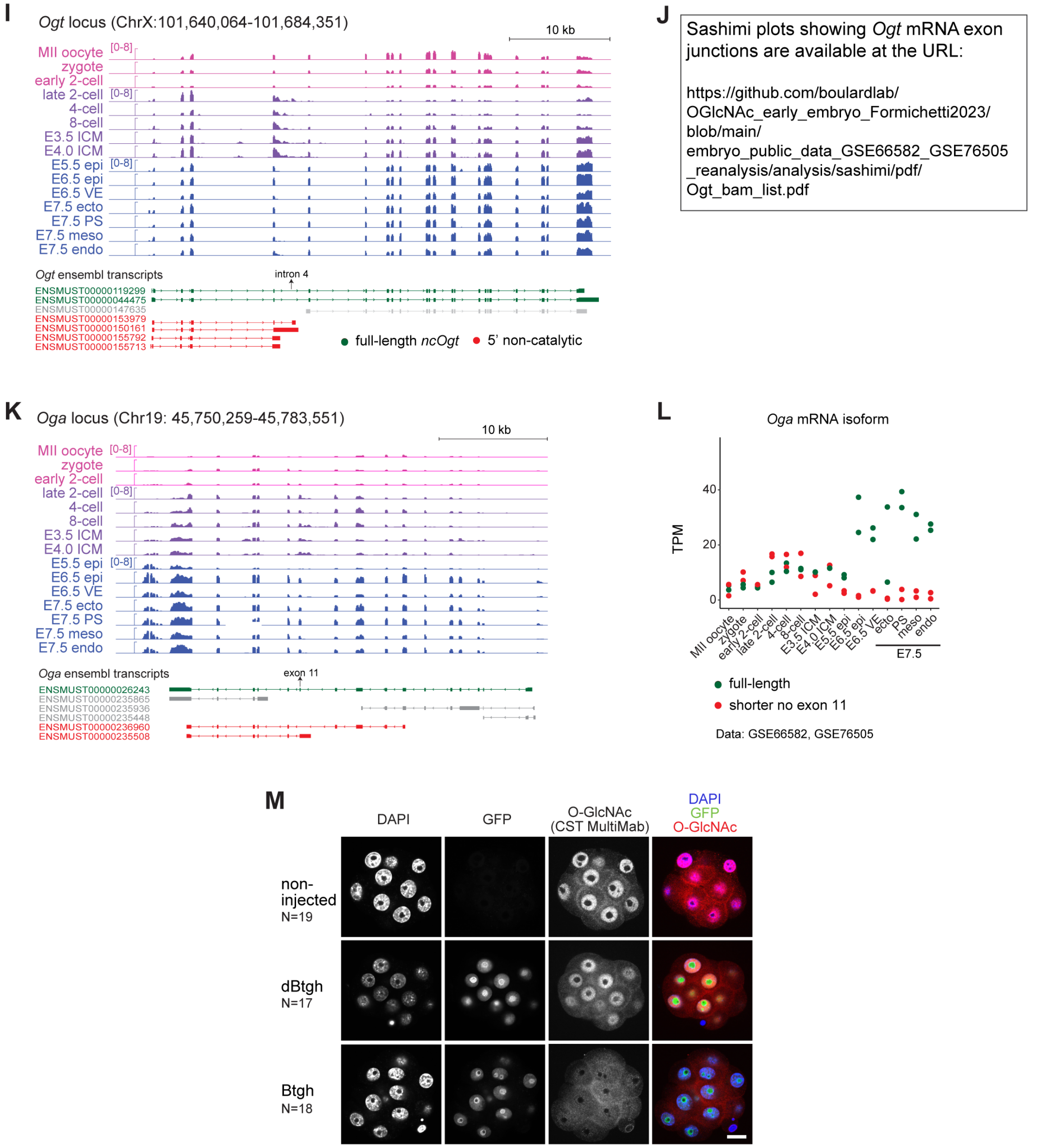

Fig. S1. Uncoupled dynamics of OGT and O-GlcNAc across mouse preimplantation development. *(continued)*

(A, B) Immunofluorescence staining of OGT **(A)** and O-GlcNAc **(B)** in zygotes at other pronuclear stages than the representative one shown in Fig. 1A and B, to give a comprehensive picture of the subcellular pattern of the enzyme and its modification across all zygotic stages. One z-plane is shown for each embryo, except for some zygotes for which two z-planes are shown. PN = pronuclear stage.
(C) Immunofluorescence staining of OGA in MII oocytes, zygotes at various pronuclear stages, 2-cell embryos (22-26 h post-IVF), morulae (72 h post-IVF) and blastocysts (96 h post-IVF) generated through IVF. One z-plane is shown for each embryo, except for some zygotes for which two z-planes are shown.

(D) Immunofluorescence staining of OGT at different zygotic pronuclear stages and in 2-cell embryos collected after natural mating throughout the day of plug and 40 h post-hCG, respectively. One z-plane is shown for each embryo, except for some zygotes for which two z-planes are shown.
(E) Immunofluorescence staining of O-GlcNAc (mix of rabbit monoclonal antibodies anti-O-GlcNAc, MultiMab CST) in MII oocytes, zygotes, 2-cell embryos (22-26 h post-IVF), morulae (72 h post-IVF) and blastocysts (96 h post-IVF) generated through IVF. One z-plane is shown for each embryo.
(**F**) Enlarged view of preimplantation nuclei from Fig. 1B and S1C (O-GlcNAc and OGA staining), to emphasize the exclusion between OGA/O-GlcNAc-rich regions and DAPI-rich regions. The scale bars indicate 10 μm.
(**G**) Immunofluorescence co-staining of O-GlcNAc (HGAC85 antibody) and OGA. Co-localization of the two signals at perinucleolar foci in both parental pronuclei is indicated by white arrows. Different z-planes are shown for the same zygote; note that the signal is visible only on certain nuclear planes. Five zygotes were imaged, with the same result.
(H) Immunofluorescence staining of the O-GlcNAc modification in 2-cell embryos from zygotes injected with Btgh or dBtgh, using a different antibody than in Fig. 2B (HGAC85) which shows signal at perinucleolar foci. Both effective O-GlcNAc removal and specificity of the O-GlcNAc signal at perinucleolar foci are confirmed by signal disappearance after Btgh injection, observed in all the 10 imaged Btgh-injected embryos. One z-plane is shown.
(I) Genome browser view of the *Ogt* gene, showing the alignment of mRNA-Seq reads from GSE66582 (1) (MII oocyte to E4.0 ICM) and GSE76505 (2) (E5.5 to E7.5). One biological replicate per stage is shown.
(J) Sashimi plot of the annotation-independent analysis of *Ogt* mRNA exon junctions from the same datasets as in Fig. 1C and *SI Appendix*, Fig. S1I. Numbers indicate the raw number of reads spanning each junction. For each stage, biological replicates are overlapped, except for the late 2-cell for which one of the two replicates was discarded because of several contaminant high peaks. Only one alternative splicing event is found, precisely inside intron 4, and only during preimplantation development from the late 2-cell stage.
(K) Genome browser view of the *Oga* gene showing the alignment of mRNA-Seq reads from GSE66582 (1), (MII oocyte to E4.0 ICM), and GSE76505 (2) (E5.5 to E7.5). One biological replicate per stage is shown.
(L) Salmon quantification of the *Oga* transcript isoforms from the same datasets as in (**K**).
(M) Confocal imaging of morulae from zygotes injected with Btgh/dBtgh or non-injected, stained with a third anti-O-GlcNAc antibody to Fig. 2B and S1H (MultiMab CST). One z-plane is shown for each embryo.
(A-E, G-H, M) Scale bar indicates 20 μm for the whole figure if present only once or for each stage if repeated.
(I-L) epi = epiblast, ecto = ectoderm, PS = primitive streak, meso = mesoderm, endo = endoderm.

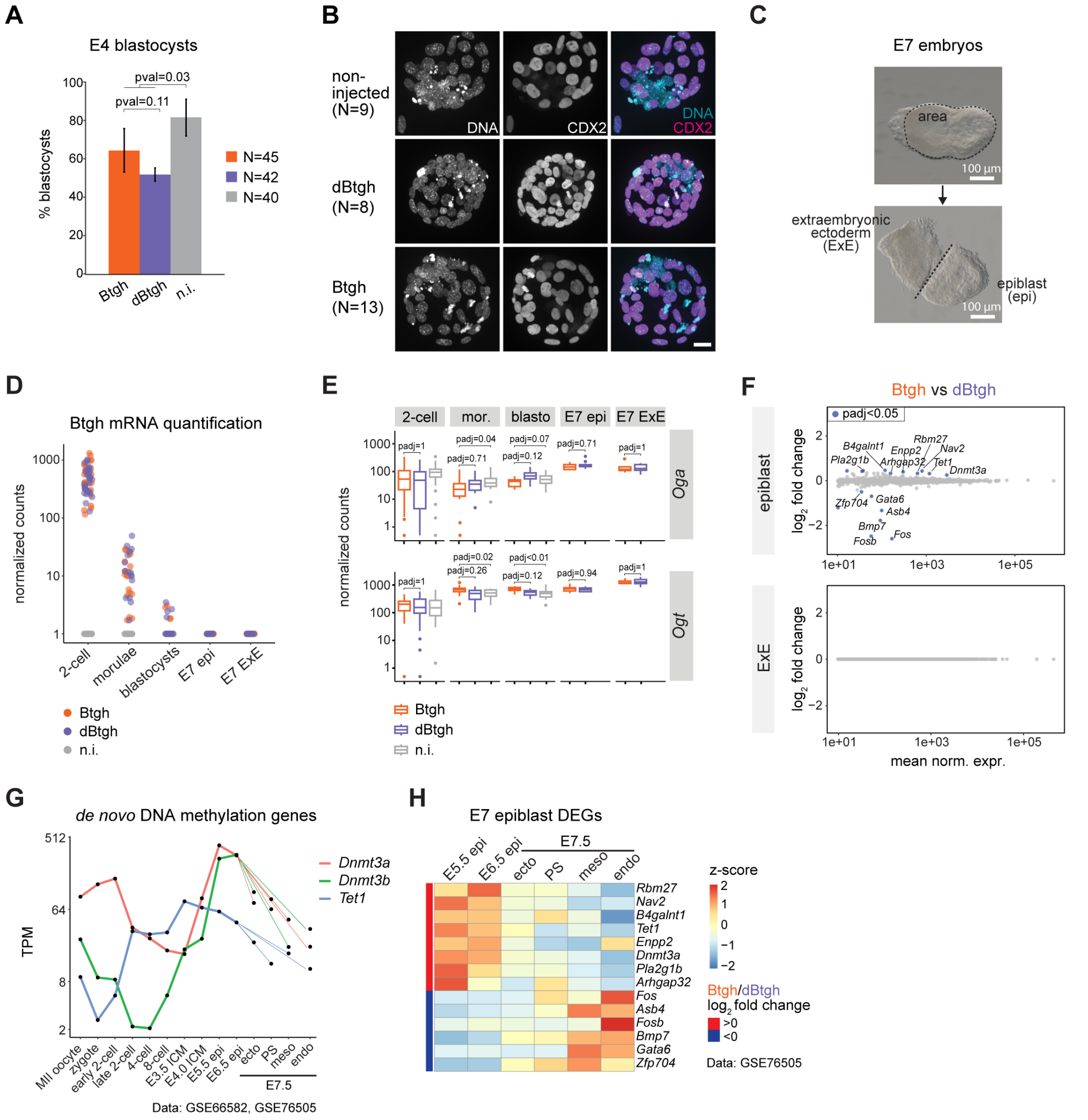

Fig. S2. Nuclear O-GlcNAc depletion does not affect differentiation but slows down development.

(A) Percentage of healthy embryonic day 4 (E4) blastocysts which developed *ex-vivo* from Btgh/dBtgh injected and non-injected zygotes. Bar heights and error bars indicate the average and standard deviation, respectively, for four replicates of the microinjection experiment. The total number of starting 2-cell embryos is stated in the figure legend. The lower developmental rate of both injected groups is expected and due to the injection procedure. The P-value was computed using unpaired Student's t-test, assuming unequal variance.

(B) Immunofluorescence staining of the trophectoderm marker CDX2 in blastocysts developed from Btgh/dBtgh-injected and non-injected embryos. The maximum projection of all z-planes is shown. Scale bar indicates 20 µm.

(C) Trans-illumination image of a dissected E7 embryo. The dash line in the top image indicates how the area was measured as a proxy of the size of the embryo (shown in Fig. 3C). The dash line in the bottom image shows the cut performed to separate the two halves, largely corresponding to the epiblast (epi) and extraembryonic ectoderm (ExE) tissues. The transcriptome of individual epi and ExE was analyzed using single-embryo mRNA-Seq.
(D) DESeq2-normalized counts of Btgh/dBtgh RNAs at all embryonic stages analyzed in this study. Y-axis ticks are in $\log_{10}$ scale.
(E) DESeq2-normalized counts of *Oga* and *Ogt* at all embryonic stages analyzed in this study, showing the compensatory downregulation of *Oga* and upregulation of *Ogt* in morulae and blastocysts upon depletion of nuclear O-GlcNAc. padj = adj. p-value computed using DESeq2 Wald test and corrected for multiple testing using the Benjamini and Hochberg method. Y-axis ticks are in $\log_{10}$ scale.
(F) MA-plots from DESeq2 differential expression analysis of E7 epiblasts or ExE from Btgh-injected embryos versus dBtgh-injected ones. Only genes with mean of DESeq2-normalized counts ≥ 10 are shown. All genes with adj. p-value < 0.05, any log2FC are colored and labeled (except three pseudogenes).
(G) Expression of *de novo* DNA methyltransferases *Dnmt3a* and *Dnmt3b* and DNA 5-methylcytosine hydroxylase *Tet1* throughout mouse embryonic development (mRNA-Seq data from GSE66582 (1) and GSE76505 (2)). The two biological replicates per stage were averaged. Y-axis shows Transcripts Per Million (TPM) and ticks are in $\log_2$ scale.
(H) Heatmap of the expression of all differentially expressed genes (DEGs; adj. p-value < 0.05, any $\log_2$FC) in Btgh-injected epiblasts (labelled in (**F**)) at the transition between E5.5 and E7.5 (mRNA-Seq data from GSE76505 (2)). TPM values were $\log_2$-transformed and scaled by rows.
(D, E, G, H) epi = epiblast, TE = trophectoderm, ecto = ectoderm, PS = primitive streak, meso = mesoderm, endo = endoderm.

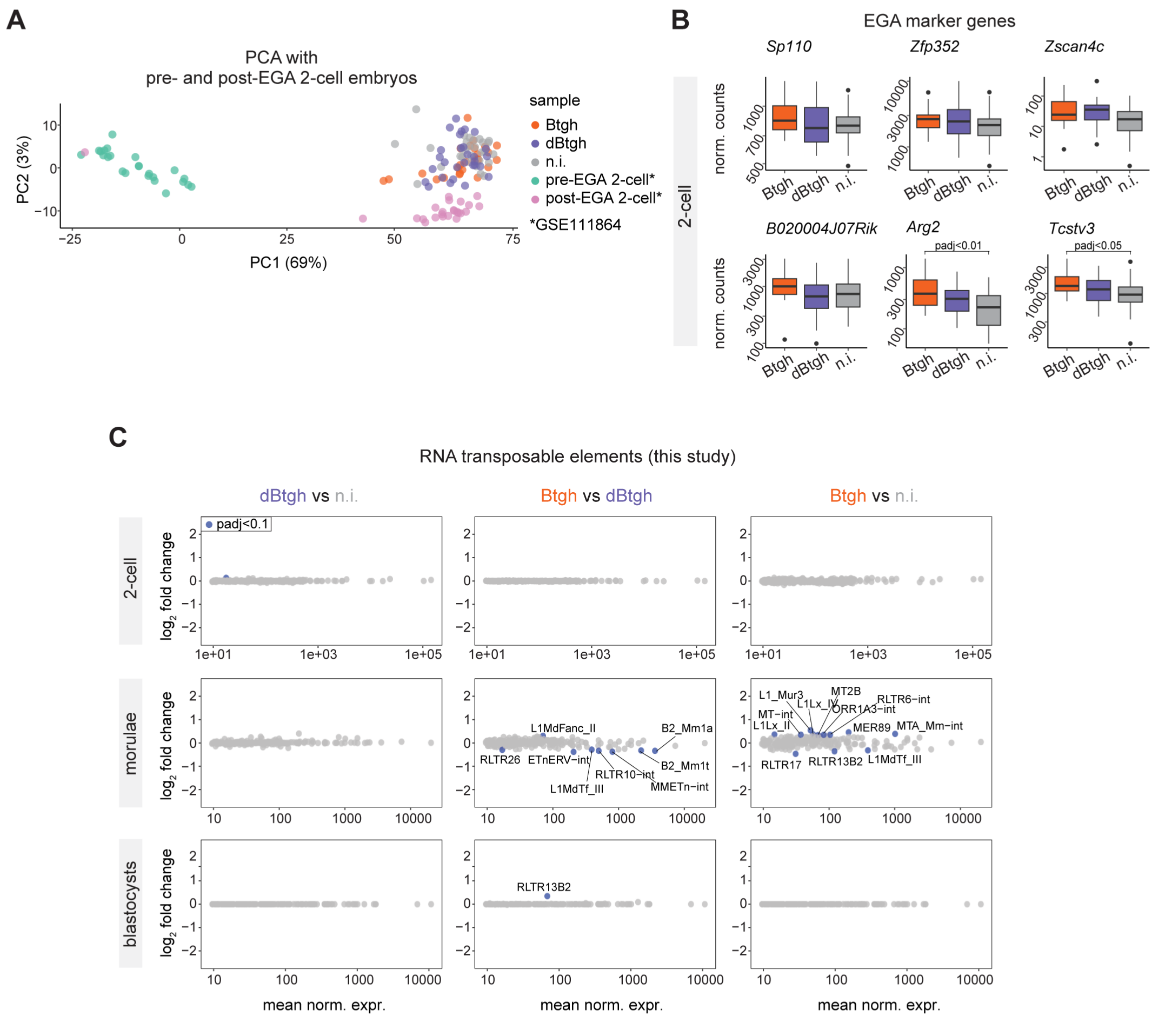

Fig. S3. Expression of retrotransposons in nuclear O-GlcNAc-depleted and unperturbed preimplantation embryos. *(continued on page 9)*

(A) PCA of the three experimental groups of 2-cell embryos from this study, together with pre-EGA and post-EGA 2-cell embryos generated through ICSI (GSE111864; ref. 3). The 200 EGA+maternal genes with the highest variance in the GSE111864 dataset were used to perform the PCA.

(B) DESeq2-normalized counts of six EGA-associated genes in the three indicated experimental groups of single 2-cell embryos. Padj = adj. p-value computed using DESeq2 Wald test and corrected for multiple testing using the Benjamini and Hochberg method, specified only when <0.05. Y-axes ticks are in $\log_{10}$ scale.

(C) MA-plots from DESeq2 differential expression analysis of RNA transposable elements between the three experimental groups of embryos at the three preimplantation stages. Only retrotransposons with mean of DESeq2-normalized counts ≥ 10 are shown. All retrotransposons with adj. p-value < 0.1, any $\log_2$FC are colored, the ones with absolute $\log_2$FC ≥ 0.2 are labelled.

D

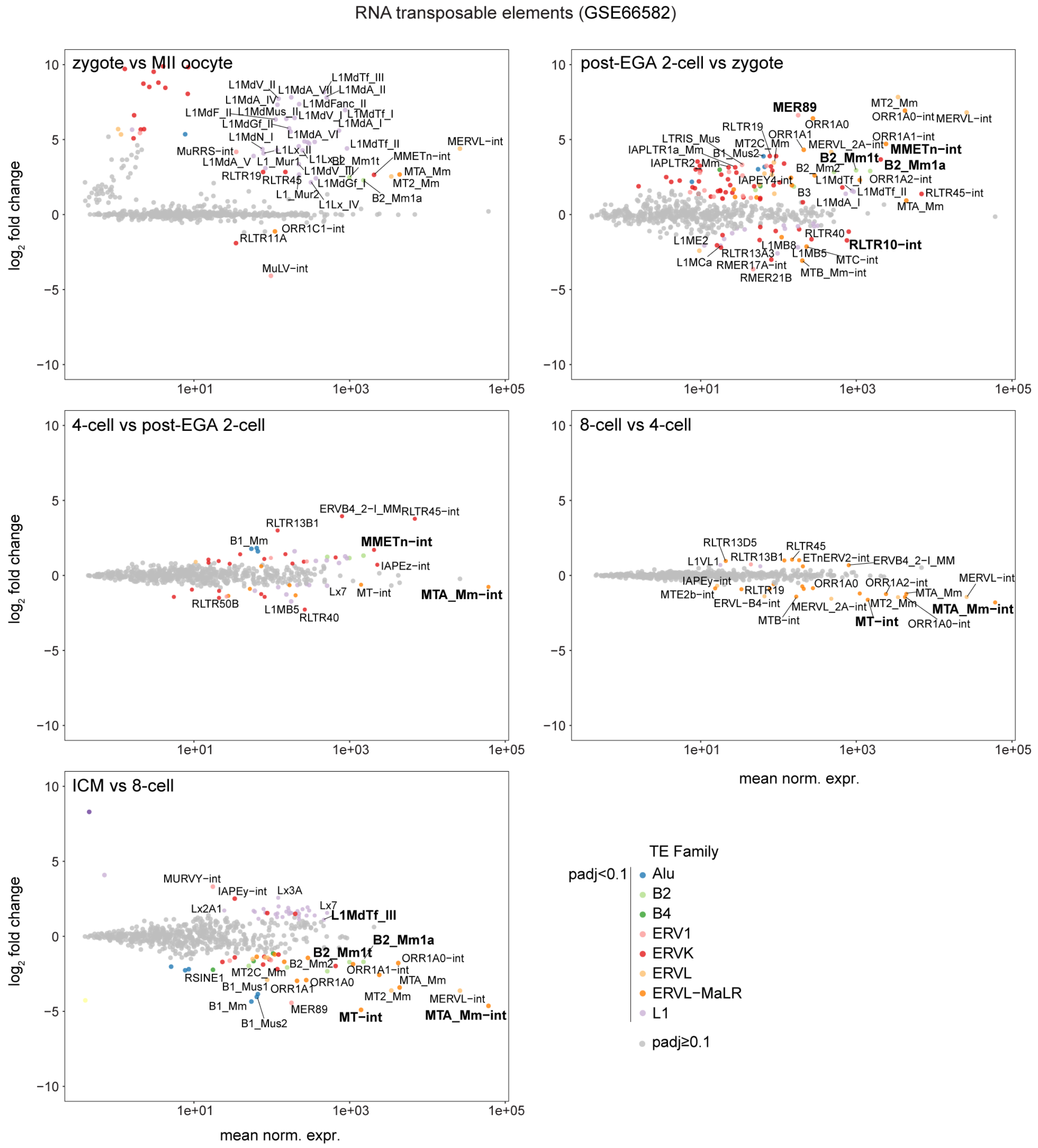

Fig. S3. Expression of retrotransposons in nuclear O-GlcNAc-depleted and unperturbed preimplantation embryos. *(continued)*

(D) MA-plots from DESeq2 differential expression analysis of RNA transposable elements between successive stages of preimplantation development in publicly available mRNA-Seq data GSE66582 (ref. 1). Pre-EGA 2-cell embryos were discarded because one replicate out of two showed high DNA contamination based on DNA transposons FPKM values. Retrotransposons with adj. p-value < 0.1 are colored by family, labeled if standing out, in bold if significantly deregulated after O-GlcNAc depletion **(B)**. Note that, with this analysis, elements which are more abundant in the genome (such as LINE elements) could be particularly sensitive to overestimated expression due to genomic DNA contamination of the RNA-Seq library.

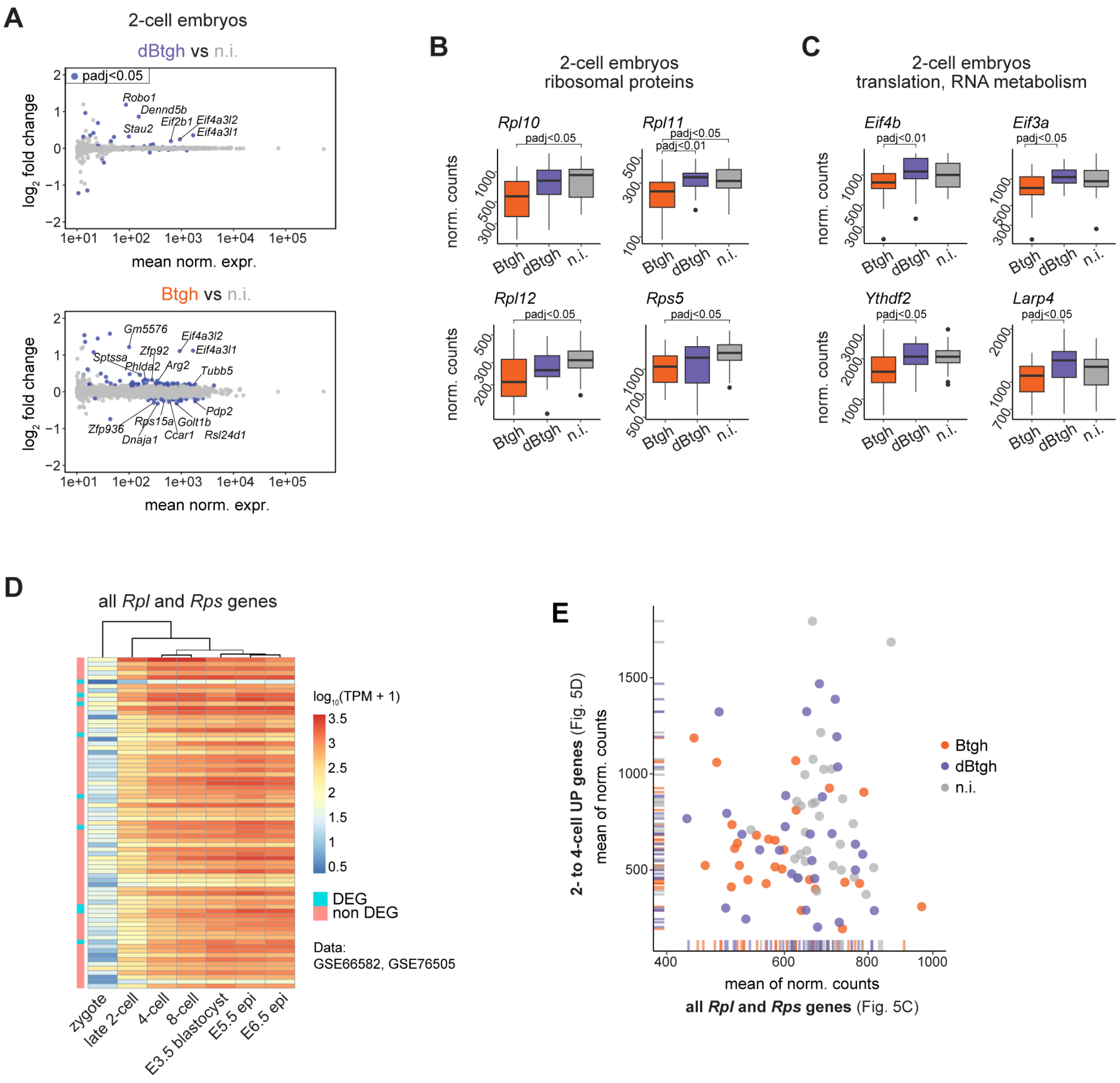

Fig. S4. Misregulation of mitotic and translation-related genes in nuclear O-GlcNAc-depleted embryos. *(legends on page 13)*

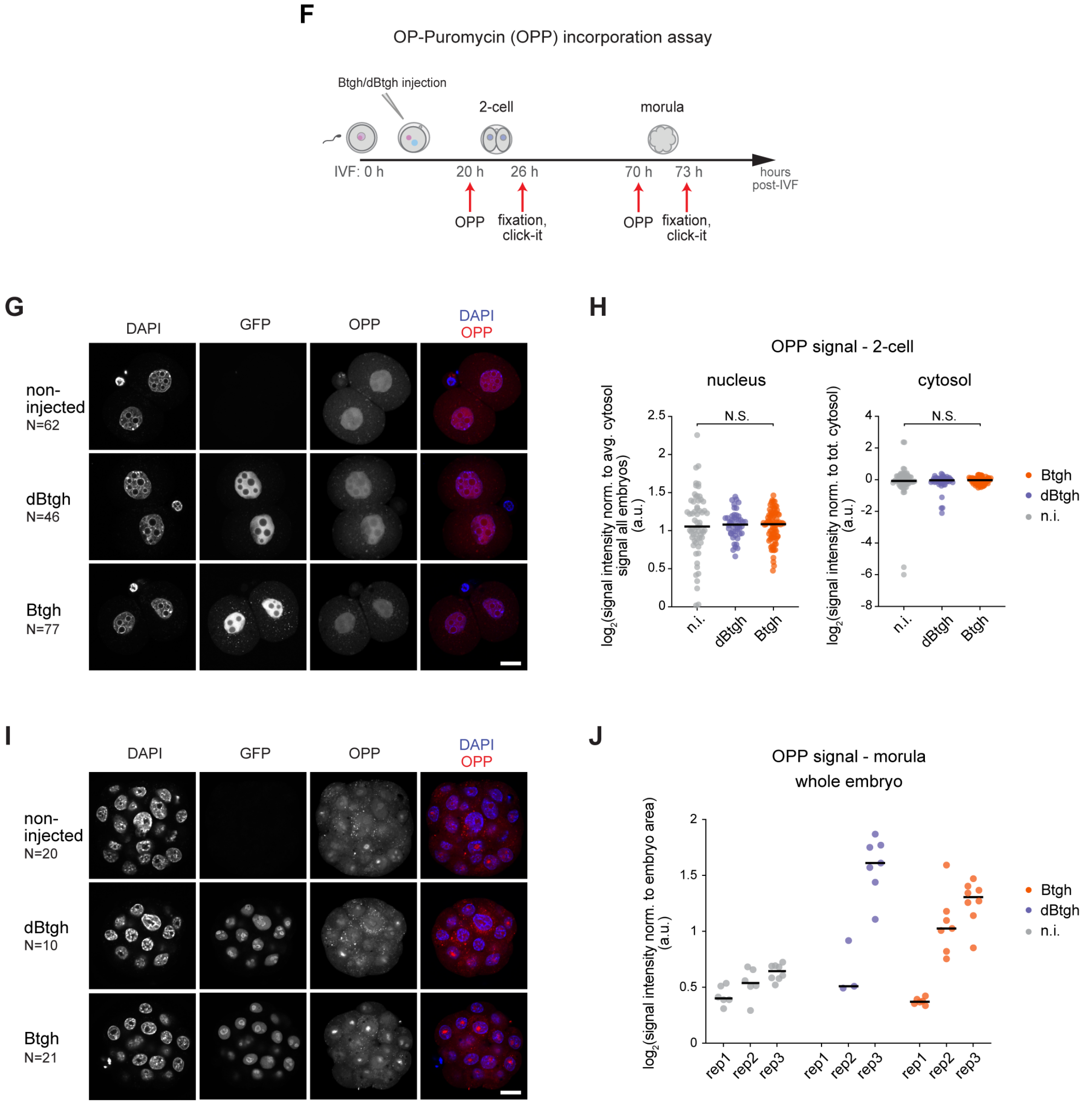

Fig. S4. Misregulation of mitotic and translation-related genes in nuclear O-GlcNAc-depleted embryos. *(continued, legends on page 13)*

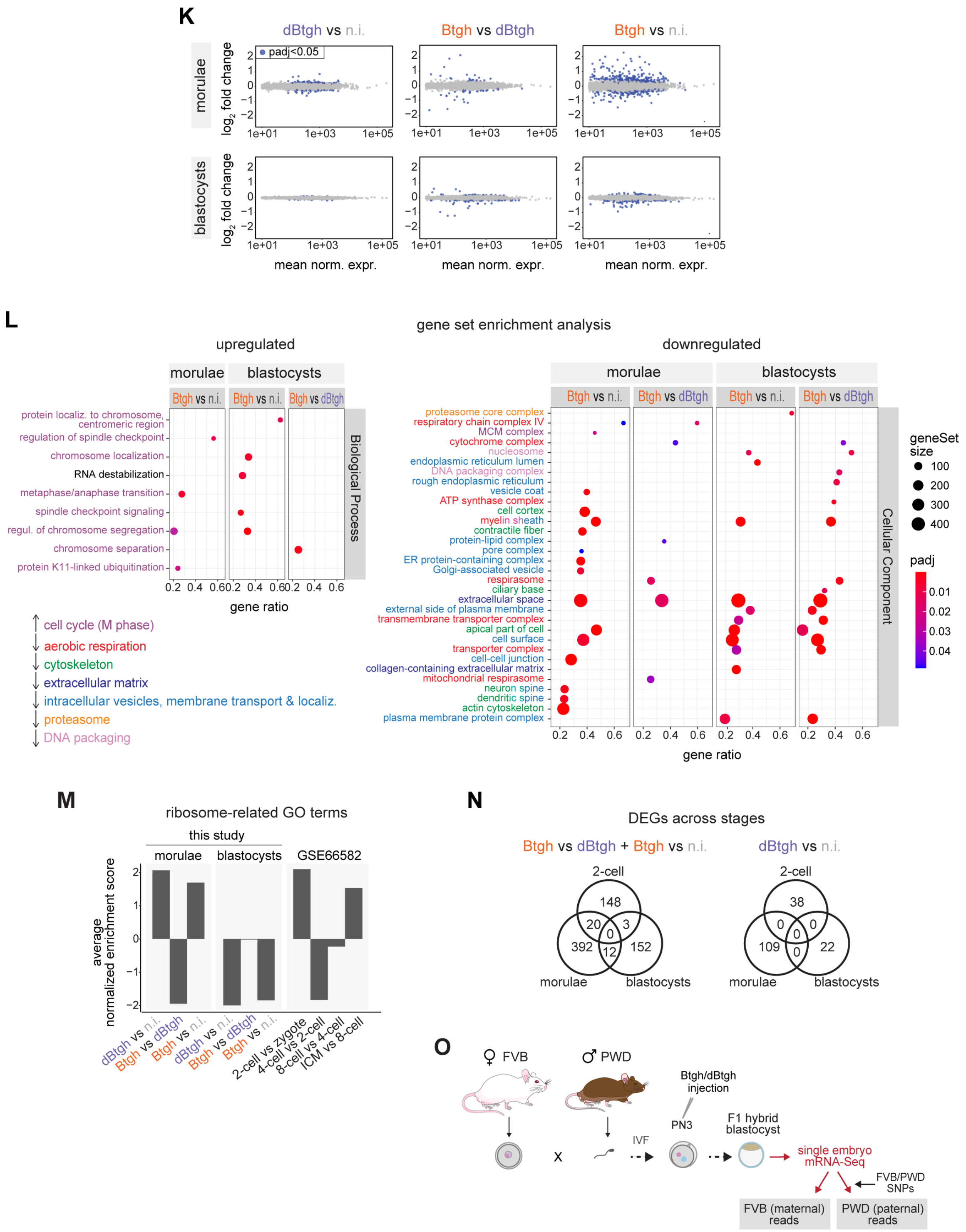

Fig. S4. Misregulation of mitotic and translation-related genes in nuclear O-GlcNAc-depleted embryos. *(continued, legends on page 13)*

Fig. S4. Misregulation of mitotic and translation-related genes in nuclear O-GlcNAc-depleted embryos. *(legends)*

(A) MA-plots from DESeq2 differential gene expression in 2-cell embryos injected by Btgh or dBtgh *vs* non-injected embryos. Only genes with mean of DESeq2-normalized counts ≥ 10 are shown. All genes with adj. p-value < 0.05, any log_2FC are colored. Genes standing out are labeled.

(B,C) DESeq2-normalized counts of (B) four ribosomal proteins and (C) genes linked to RNA metabolism significantly downregulated in 2-cell embryos after nuclear O-GlcNAc depletion. padj = adj. p-value computed using DESeq2 Wald test and corrected for multiple testing using the Benjamini and Hochberg method.

(D) Heatmap of TPM values for all *Mus musculus* ribosomal protein genes from the Ribosomal Protein Gene Database (4). Both rows and columns are clustered based on Spearman correlation. Genes which are differentially regulated at any stage after nuclear O-GlcNAc depletion (DEGs) are indicated.

(E) Average expression of all ribosomal protein genes (Fig. 5C) versus average expression of genes upregulated between the 2- and 4-cell stage (Fig. 5D) in single embryos from the three experimental groups. One outlier non-injected embryo was excluded.

(F) Experimental design for results in **(G-J)**. Zygotes were generated through IVF and injected 2 h later with the mRNA of Btgh or dBtgh or left uninjected. The three groups of embryos were cultured ex-vivo until the 2-cell stage or the morula stage and then incubated with the OPP reagent for 6 h and 3 h, respectively for each stage, to monitor protein translation during these two time windows. At the end of the incubation, embryos were fixed and the Click-It reaction performed, which allowed fluorescence imaging of the signal of the incorporated OPP.

(G,I) Immunofluorescence imaging of the 26 h 2-cell embryos **(G)** and the morulae **(I)** from the experiment in **(F)**. Scale bar indicates 20 µm for the whole figure. The GFP signal comes from the Btgh/dBtgh protein fused to GFP.

(H,J) Quantification of OPP signal intensity from **(G)** and **(I)**, performed as described in Methods. For the 2-cell embryos **(G)**, p-value was computed using one-way ANOVA with post-hoc Tukey HSD and it was > 0.05 thus considered non-significant. For the morulae **(J)**, the three replicates of the IVF-microinjection-OPP experiment are plotted separately to show the incoherent (i.e. non-reproducible) trend among replicates of the OPP signal's change between Btgh-injected and dBtgh-injected embryos.

(K) MA-plots from DESeq2 differential gene expression analysis between experimental groups of morulae and blastocysts. Only genes with mean of DESeq2-normalized counts ≥ 10 are shown. All genes with adj. p-value < 0.05, any log_2FC are colored, and their number is indicated.

(L) Gene set enrichment analysis of gene expression changes in nuclear O-GlcNAc-depleted morulae and blastocysts versus controls. Among the significant gene ontology (GO) terms, the upregulated Biological Process (BP) terms and the downregulated Cellular Component (CC) terms with the highest Normalized Enrichment Score are shown, ordered by gene ratio. Downregulated BPs and upregulated CCs can be found at:
https://boulardlab.github.io/OGlcNAc_early_embryo_Formichetti2023/reports/Btgh_injected_blastocysts_SMARTSeq/GSEA_morulae_blasto_comparison.html. Ribosome-related terms were excluded and plotted in **(M)**. The size of dots is proportional to the number of total genes of a GO term. Gene ratio = fraction of total genes of the GO term which are concordantly changing between the two conditions.

(M) Average Normalized Enrichment Score for all ribosome- and translation-related terms found among significant terms in the GSEA results of (left) the morula and blastocyst datasets produced in this study and (right) a publicly available mRNA-Seq dataset spanning mouse preimplantation stages (GSE66582). Two-cell indicate post-EGA 2-cell embryos.

(N) Overlap among differentially expressed genes (DEGs; adj. p-value < 0.05, any log_2FC) in Btgh-injected embryos versus any of the control groups (left) and in dBtgh-injected versus non-injected embryos (right) at the different preimplantation stages. DEGs were included only if DESeq2-normalized counts ≥ 10 at the corresponding stage.

(O) Experimental scheme to produce F1 hybrid (FVB/PWD) embryos to assess whether depletion of nuclear O-GlcNAc affects paternal/maternal autosomal ratio as a measure of proper chromosome segregation.

	PS	PS/ES	ES	advanced ES	ES or MS	MS	Not determined	Tot determined	Pre-ES %	ES %	Adv./ Post-ES %
**Btgh**	2	1	8	0	2	2	9	15	13	60	27
**dBtgh**	2	4	7	2	1	4	11	20	10	55	35

Table S1. Staging of E7 embryos based on widefield microscopy images.

Two-way Anova p-value = 0.2

PS = Primitive Streak, ES = Early Streak, PS/ES = between PS and ES, advanced ES = advance Early Streak, MS = Mid Streak, Not determined = staging uncertain.

Sequence type	Target	Primer direction	Sequence (5'-3')
Primer	Btgh-containing plasmid (Boulard *et al*., 2020)	forward	GGAAGCATCAGGCGGGCTGCAGAATAGTAAAGGAGAA GAACTTTTCACTGGAGTTGTCCCA
		reverse	GAGAAACATTCTGTCCGCCTTTGTATAGTTCATCCATGC CATGTGTAATCC
Primer	NLS-mgfp5 gBlock	forward	CAAAGGCGGACAGAATGTTTCTCTCCAACCTCCAC
		reverse	AGTGGTAACCAGATCCGCTCACACTTTCCGTTTCTTCTT AGGATCG
NLS-mgfp5 gBlock			GATCTGAATTCCTGCAGCCCGCCACCATGGCCTCTCCTA AGAAAAAGAGGAAAGTGGAAGCATCAGGCGGGCTGCA GAATAGTAAAGGAGAAGAACTTTTCACTGGAGTTGTCC CAATTCTTGTTGAATTAGATGGTGATGTTAATGGGCACA AATTTTCTGTCAGTGGAGAGGGTGAAGGTGATGCAACA TACGGAAAACTTACCCTTAAATTTATTTGCACTACTGGA AAACTACCTGTTCCATGGCCAACACTTGTCACTACTTTC ACTTATGGTGTTCAATGCTTTTCAAGATACCCAGATCAT ATGAAGCGGCACGACTTCTTCAAGAGCGCCATGCCTGA GGGATACGTGCAGGAGAGGACCATCTTCTTCAATGACG ACGGGAACTACAAGACACGTGCTGAAGTCAAGTTTGAG GGAGACACCCTCGTCAACAGGATCGAGCTTAAGGGAAT CGATTTCAAGGAGGACGGAAACATCCTCGGCCACAAGT TGGAATACAACTACAACTCCCACAACGTATACATCATG GCCGACAAGCAAAAGAACGGCATCAAAGCCAACTTCAA GACCCGCCACAACATCGAAGACGGCGGCGTGCAACTCG CTGATCATTATCAACAAAATACTCCAATTGGCGATGGCC CTGTCCTTTTACCAGACAACCATTACCTGTCCACACAAT CTGCCCTTTCGAAAGATCCCAACGAAAAGAGAGACCAC ATGGTCCTTCTTGAGTTTGTAACAGCTGCTGGGATTACA CATGGCATGGATGAACTATACAAAGGCGGACAGAATGT TTCT

Table S2. Primers and gBlocks used for cloning the pRN3P-NLS-EGFP-Btgh/dBtgh-3xNLS plasmids.

Stage	# pooled embryos in sequencing run	Average # of reads per embryo	# pooled embryos in sequencing run, per group			# embryos discarded for < 10^6 reads	# embryos discarded for high rDNA/mt gene counts	# embryos discarded for low Btgh expression	# embryos discarded for opposite tissue contamination	# embryos discarded for aneuploidy	# embryos for Diff. Expr. analysis
2-cell	96	$5.3* 10^6$	Btgh		32	3	1	1	Not applicable	Not assessed	27
			dBtgh		32	2	0	1	Not applicable	Not assessed	29
			n.i.		32	3	0	0	Not applicable	Not assessed	29
morula	62	$6.3* 10^6$	Btgh		22	1	0	3	Not applicable	Not assessed	18
			dBtgh		18	1	0	1	Not applicable	Not assessed	16
			n.i.		22	0	0	0	Not applicable	Not assessed	22
blastocyst	62	$6.1* 10^6$	Btgh		19	0	2	0	Not applicable	3	14
			dBtgh		20	0	1	0	Not applicable	2	17
			n.i.		23	4	0	0	Not applicable	2	17
E7	25 epi + 26 ExE	$10.5* 10^6$	Btgh	epi	13	1	0	0	0	0	12
				ExE	13	1	0	0	2	0	10
			dBtgh	epi	12	0	0	0	0	0	12
				ExE	13	0	0	0	0	0	13

Table S3. Details on the generation and filtering steps of the single embryo Smart-Seq datasets.

Gene target	Primer direction	Sequence (5'-3')
Xist (cDNA)	forward	TCTATCTTGTGGGTCCTGGAG
	reverse	CTCCTCTAAATCCAGGCAATCC
Ddx3y (cDNA)	forward	TGGAGGAGGAAATACAGAGAGC
	reverse	GGAGGACAATTATTTCCAGTTGC
Eif2s3y (cDNA)	forward	TGGCTGTGAAGTTGATGACC
	reverse	CCTTCTGTACGTACACCTAGG

Table S4. Primers used for PCR genotyping of E7 Btgh/dBtgh-injected embryos' cDNA.

Supplemental Methods

Animal care and strains

All procedures involving mice were handled in compliance with the rules and regulations of the EMBL Institutional Animal Care and Use Committee (IACUC) under protocol number 21-012_RM_MB, as well as with Italian Law (DL 26/2014, EU 63/2010) under protocol number 598/2023-PR to M.B. (Ministero della Sanità, Roma, Italy). Mice were housed in the pathogen-free Animal Care Facility at EMBL Rome on a 12-hours light-dark cycle in temperature and humidity-controlled conditions with *ad libitum* access to food and water. Female FVB/NCrl were used throughout the study; male FVB/NCrl were used for all imaging and NGS experiments except for the blastocyst and E7 Smart-Seq experiments, for which PWD/Ph males were used.

In vitro transcription of NLS-EGFP-Btgh-3xNLS Btgh mRNA

The cDNA encoding O-GlcNAc hydrolase (*Oga*, *Btgh84*) from Bacteroides was amplified from the plasmid described in Boulard *et al.* (5) and cloned in the pRN3P-H3.3R8A-GFP plasmid using restriction enzymes NotI and SmaI, fused to enhanced GFP *mgfp5* and flanked by SV40 NLSs. pRN3P-H3.3R8A-GFP is a generous gift from Maria Elena Torres-Padilla (6). The catalytically inactive ("dead") *Btgh84*^{D242A} was cloned the same way (7). Sequences of primers and synthetic double-stranded DNA (IDT) used for plasmids cloning are in *SI Appendix*, Table S2.

The plasmid was linearized by digestion with KpnI, purified and then used as a template for mRNA synthesis with mMESSAGE mMACHINE T3 (Invitrogen #AM1348). In vitro transcribed RNA was purified with RNA Clean & Concentrator Kit (Zymo Research) and eluted in H_2O. The mRNA product was run on an RNA ScreenTape TapeStation 4150 (Agilent) to verify the presence of one main band of the correct size, the concentration was measured with NanoDrop 2000 spectrophotometer (ThermoFisher) and working concentration aliquots were made by diluting the mRNA in H_2O. Aliquots were stored at -70 °C until microinjection.

In vitro fertilization (IVF)

Superovulation of 6–8 weeks FVB females was induced by hormonal stimulation (5 IU of PMSG and 5 IU of hCG 64 h and 16 h before collection, respectively), and cumulus-oocyte complexes were collected in KSOM media containing GSH (final concentration 10 mM; Sigma-Aldrich #G6013). Concomitantly, cauda epididymis and vasa deferentia from FVB or PWD males were dissected and sperm was gently squeezed out into capacitation media (HTF supplemented with MBCS at final concentration of 0.75 mM) (HTF: Sigma-Aldrich #MR-070-D; MBCD: Sigma-Aldrich #C4555), and allowed to swim up for 1 hour. Sperm was subsequently counted and cumulus-oocyte complexes were inseminated with 0.2M sperm in a 200 µL fertilization drop. Four hours after sperm addition, zygotes were cleaned from the surrounding cumulus cells and sperm by 5–6 washes in KSOM, then cultured in KSOM in a standard mammalian cell incubator.

Immunofluorescence staining of preimplantation embryos

After two washes in M2 medium, the zona pellucida of the embryos was removed by a brief incubation in drops of warm Acidic Tyrode's solution (Sigma-Aldrich), followed by other two M2 washes to neutralize the acid. The embryos were then washed once in PBS + 0.5% BSA (Bovine Serum Albumin, Sigma-Aldrich #A2153), fixed in 4% PFA (Thermo Scientific #28908) in PBS for 20 min at 37 °C, permeabilized in 0.5% Triton X-100 (Sigma-Aldrich #X100-100ML) for 20 min at 37 °C and washed

three times in PBS-T (0.15% Tween-20 in PBS). The epitope was then unmasked in 50 mM NH_4Cl solution in H_2O for 10 min at room temperature, followed by two additional PBS-T washes and then blocking — for 3 h at room temperature or overnight at 4 °C — in BSA 3% in PBS-T. Primary antibody incubation was performed overnight at 4 °C in the blocking solution, followed by three washes in PBS-T, re-blocking for 30 min at room temperature, three additional PBS-T washes, secondary antibody incubation for 1 to 2 h at room temperature in the blocking solution and three final PBS-T washes, after which the embryos were mounted as described in the Immunofluorescence Methods section.

Single embryo Smart-Seq protocol

The plate containing frozen single embryo's mRNA was then sent to EMBL Gene Core Facility, which used a modified Smart-Seq2 protocol (8) using SuperScript IV RT and tagmentation procedure previously described (9) to prepare single-embryo full-length cDNA sequencing libraries. The retrotranscription reaction mix was as follows: 2 µL SSRT IV 5x buffer, 0.5 µL 100 mM DTT, 2 µL 5 M betaine, 0.1 µL 1 M MgCl2, 0.25 µL 40 U/µL RNAse inhibitor, 0.25 µL SSRT IV, 0.1 µL 100 uM TSO, 1.15 µL RNase-free H_2O; with thermal conditions: 52 °C 15 min, 80 °C 10 min. cDNA was generated using 18 PCR cycles. The cDNA cleanup (0.6x SPRI ratio; SPRIselect beads: Beckman Coulter #B23319) was carried out omitting the ethanol wash steps and the elution volume was 13 µL of H_2O. For tagmentation, the sample input was normalized to 0.2 ng/uL. After tagmentation and PCR, 2 µL of each sample were pooled in a single tube before the final clean up using 0.7x SPRI ratio. The pool was sequenced in one run (40 bp paired-end mode) on the Illumina NextSeq 500 sequencer. See *SI Appendix*, Table S3 for the number of pooled embryos and average number of reads obtained per embryo at each stage.

OP-Puromycin (OPP) incorporation assay

Total protein synthesis rate was assessed by O-propargyl-puromycin (OPP) incorporation in non-injected and Btgh/dBtgh-injected embryos (Click-iT Plus OPP Alexa Fluor 647, Thermo Scientific #C10457) in 2-cell embryos (20 h post-IVF) and morulae (70 h post-IVF) with adaptations from the manufacturer assay and from Li *et al.* (10). IVFs and zygote microinjections were performed as detailed in the corresponding methods section. Briefly, embryos were incubated in microdrops of KSOM supplemented with 25 µM OPP reagent, which had been dissolved in DMSO (Sigma-Aldrich #D6650) and added to KSOM media at 0.25% (v/v) final DMSO concentration in KSOM. The drops were overlaid with mineral oil (Sigma-Aldrich #M8410) and pre-equilibrated for 30 minutes in the incubator (5% CO2, 37 °C). 2-cell stage embryos were added to the drops and incubated for 6 h (26 h post-IVF), while morulae for 3 h (73 h post-IVF). Embryo density in the micro drops was the same for all conditions.

After OPP incubation, the embryos were washed once in 3% BSA (Sigma-Aldrich #A7906) in PBS and fixed in 4% PFA in PBS for 20 min at 37 °C, then washed twice in 3% BSA in PBS, permeabilized in 0.5% Triton X-100 in PBS for 20 minutes at room temperature and washed twice in 3% BSA in PBS. For the Click reaction, embryos were incubated in the Click-iT reaction cocktail for 30 minutes, protected from light. Finally, the embryos were washed in the Click-iT reaction rinse buffer, mounted in Vectashield with DAPI, and imaged using a Nikon AX scanning confocal with a 60x objective. Acquisition of each embryo was performed as a Z stack with 1 µm steps.

Quantification of the OPP signal

To quantify the incorporation of OPP in nascent proteins, the fluorescent intensity of OPP (Alexa 647) in the nucleus and cytoplasm (2-cell) or whole embryo (morulae) was measured with ImageJ. For the 2-cell stage embryos, DAPI staining was used to create a mask to the single nuclei, then quantification of the nuclear and cytosolic OPP signal was performed in single blastomeres and normalized to the cytoplasmic average intensity level of all embryos of that experimental replicate (N=2). For the morulae, the total OPP-signal intensity of each morula was normalized to individual embryonic areas (dBtgh, N=2; non-injected, aBtgh, N=3). For the 2-cell embryos (*SI Appendix*, Fig. S4H), data from each replicate of the IVF-microinjection-OPP experiment were pooled in one single plot, log2-transformed and the comparison between conditions statistically assessed by one-way ANOVA (ANalysis Of VAriance) with post-hoc Tukey HSD (Honestly Significant Difference). Less than a 5% probability ($P < 0.05$) was considered significant. For the morulae (*SI Appendix*, Fig. S4J), the three replicates of the IVF-microinjection-OPP experiment were plotted separately to show the incoherent trend among replicates of the OPP signal's change between Btgh-injected and dBtgh-injected embryos, i.e. a non-reproducible up- or down-regulation of protein synthesis upon O-GlcNAc removal. For this experiment, statistical analyses were performed using GraphPad Prism version 10.3.1 (GraphPad Software).

Single embryo Smart-Seq data analysis for single copy genes

Except for allele-specific analysis (in corresponding SI Methods section), the analysis pipeline was performed using Galaxy (11) to obtain the table of gene counts (except for allele-specific analysis, detailed in *SI Appendix*). Briefly, the quality of the reads was analyzed using FastQC v0.11.8 (https://www.bioinformatics.babraham.ac.uk/projects/fastqc/). Reads were trimmed from adapters and low-quality 3'-end nucleotides using Trim Galore v0.6.3 (https://www.bioinformatics.babraham.ac.uk/projects/trim_galore/) with default parameters for paired-end libraries (-q 20 --stringency 1 -e 0.1 --length 20 –paired), before mapping them using STAR v2.7.8a (12) and default parameters for paired-end reads to the GRCm38 mouse genome containing the NLS-GFP-Btgh-3xNLS transgene. Gene counts were obtained from the bam files using featureCounts (subreads v2.0.1) (13), with default parameters for paired-end reads and counting fragments instead of reads. The gene counts were used in a custom Rmd script as input for DESeq2 v1.34.0 (14). The test used for statistical significance was the Wald test and the significance cut-off for optimizing the independent filtering was 0.05.

For the 2-cell embryo and blastocyst datasets, which were the result of more than one embryo generation and collection, batch effect had to be considered when testing for differential expression using DESeq function. To this aim, package RUVSeq (15) was used to compute the factors of unwanted variation, with function 'RUVs' and k=3. The 3 factors of unwanted variation computed with RUVSeq were used in 'DESeq' formula ~ *W1 + W2 + W3 + condition*.

Before PCA and differential expression analysis, a few filtering steps were applied to all datasets: embryos with $< 10^6$ reads and outlier embryos in a scatterplot of mitochondrial DNA (mtDNA) gene expression versus percentage of reads mapping to ribosomal DNA (rDNA) were removed as low-quality samples. In the blastocyst dataset, the outliers in Fig. 5G (aneuploid embryos) were additionally removed; for E7, those halves showing contamination from the complementary tissue based on the expression of markers for epiblast (*Map7d3*, *Pdzd4*, *Uchl1*) and extraembryonic ectoderm (*Gjb3*, *Gm9*, *Wnt6*) were excluded from all analyses. The number of embryos filtered at each step is in *SI Appendix*, Table S3.

Analysis of publicly available mRNA-Seq data

For the analysis of datasets GSE66582 (1) and GSE76505 (2), both transcript counts and bigwig files were obtained using Galaxy (11). In detail, the quality of the reads was analyzed using FastQC, then reads trimmed from adapters and low-quality 3'-end nucleotides using Trim Galore v0.6.3, first with default parameters and automatic adapter detection, then by specifically removing Clontech SMART CDS Primer II A 25 nt sequence (5'-AAGCAGTGGTATCAACGCAGAGTAC-3') from both forward and reverse reads. For gene expression analysis, reads were mapped to Gencode vM25 (GRCm38.p6) transcript sequences using Salmon v0.8.2 (16) with default parameters for unstranded paired-end libraries. The output transcript counts were used in a custom Rmd script as input for gene-level summarization using tximport (17). Gene-level TPM values were extracted from tximport 'abundance' matrix. To find genes upregulated between late 2-cell and 4-cell embryos (Fig. 5D) we used DESeq2; the test used for statistical significance was the Wald test, the significance cut-off for optimizing the independent filtering was 0.05 and the $\log_2$ fold changes were shrunken using the 'ashr' method (18). Then, genes were selected if: i. 2-cell TPM > 1; ii. 4-cell TPM > 3rd quartile of expression among genes with TPM > 1; iii. 4-cell/2-cell log2FC > 2. The only *Rpl* gene selected by these criteria was excluded.

Sashimi plots

The sashimi plots (*SI Appendix*, Fig. S1J) were generated using ggsashimi (19) with density overlay between biological replicates but aggregation of junction counts (parameters --aggr median_j --min-reads-coverage 3).

Principal component analysis (PCA) of single embryo transcriptomes

All included PCAs were performed with function 'prcomp' on $\log_2$-transformed and DESeq2-normalized raw counts and only genes with mean DESeq2-normalized expression > 10 across the 'prcomp' input samples were used. For *SI Appendix*, Fig. S3A, among the EGA+maternal genes (defined in the Results section), the 200 genes with the highest variance in GSE111864 were used to perform the PCA. For Fig. 4B, all DEGs (adj. p-value < 0.05, any $\log_2$FC) from the Btgh versus dBtgh and Btgh versus non-injected comparison at any stage were used.

Gene set enrichment analysis (GSEA)

Performed in a custom Rmd script using function 'gseGO' of R package 'clusterProfiler' (20) on all genes of the dataset with DESeq2-normalized mean across samples > 10, ranked by -log10(adj. p-value)*sign($\log_2$FC). P-value cutoff was 0.05 and parameters set to default. Results were simplified based on adj. p-value after computing semantic similarity using 'mgoSim' function of R package 'GoSemSim'(21). Similarity cutoff was 0.6 for preimplantation datasets, 0.8 for the E7 epiblasts. For each figure, the first significant terms based on Normalized Enrichment Score (NES) are shown, ordered by GeneRatio.

Analysis of transposable elements expression

The analysis was performed using a custom Snakemake v5.9.1 pipeline (22), available at the GitHub repository linked below. In summary, quality of the fastq files was checked with FastQC v0.11.8 and reads were trimmed using Trim Galore v0.6.4 with default parameters (-q 20 --stringency 1 -e 0.1 --length 20 –paired). Trimmed reads were aligned to GRCm38 mouse genome with STAR v2.7.5c (12), with parameters recommended by Teissandier *et al.* (23) for the analysis of transcripts derived from autonomous retrotransposons in the mouse genome: --outFilterMultimapNmax 5000 --

outSAMmultNmax 1 --outFilterMismatchNmax 3 --outMultimapperOrder Random --winAnchorMultimapNmax 5000 --alignEndsType EndToEnd --alignIntronMax 1 --alignMatesGapMax 350 --seedSearchStartLmax 30 --alignTranscriptsPerReadNmax 30000 --alignWindowsPerReadNmax 30000 --alignTranscriptsPerWindowNmax 300 --seedPerReadNmax 3000 --seedPerWindowNmax 300 --seedNoneLociPerWindow 1000. After alignment, using a custom script, we only kept reads from pairs where both mates were: i. completely included into a repetitive element; ii. not overlapping with gene bodies of Gencode vM25. Repeat Library 20140131 (mm10, Dec 2011) was used as repetitive elements' annotation, after excluding "Simple repeats" and "Low complexity repeats".
With the STAR parameters above, only one random alignment (the one with the highest alignment score) is reported for multimappers, preventing the precise quantification of single repetitive elements. Therefore, for each repName of the repetitive element annotation, often present in multiple copies in the genome, read counts were summarized using featureCounts (subreads v2.0.1) (13), with parameters -p -B -s 0 --fracOverlap 1 -M.

The downstream analysis of transposable elements' expression was performed in a custom Rmd script. First, an analysis at family level (repFamily field in the Repeat Library annotation) was performed to identify samples with an important contamination from genomic DNA, since the latter can affect the quantification of retrotransposon transcripts. For this analysis, FPKM were computed for each repFamily and samples with high FPKM values for DNA transposon families were removed. Then, differential Expression of RNA transposons was tested at the RepName level, using DESeq2 v1.34.0 (14) and adding the value of the total sum of DNA FPKM as a confounding factor to DESeq formula (~ *DNA_FPKM + condition*). The test used for statistical significance was the Wald test, and the significance cutoff for optimizing the independent filtering was 0.05.

Allele-specific Smart-Seq data analysis

The analysis was performed using a custom Snakemake v5.9.1 pipeline (22), available at the GitHub repository linked below. Firstly, the position and identity of all annotated Single Nucleotide Polymorphisms (SNPs) between the FVB/NCrl and PWD/Ph mouse strains was collected from GenomeMUSter, a resource of the Mouse Phenome Project (MPD, RRID:SCR_003212) (24). The PWD genome wasis not included in the Mouse Genome Project, making the accuracy of SNPs annotation highly variable compared to the better annotated FVB strain. Therefore, only SNPs with confidence level = 1 for the FVB DNA base and ≥ 0.8 for the PWD DNA base were kept for all analyses and used to create a masked GRCm38 mouse genome with 'maskfasta' from the BEDTools suite (25). After quality check with FastQC v0.11.8 and trimming using Trim Galore v0.6.4 with default parameters (-q 20 --stringency 1 -e 0.1 --length 20 –paired), reads were mapped to the masked genome using STAR v2.7.5c (12). Alignments parameters were the default ones, except for --alignEndsType EndToEnd and --outSAMattributes NH HI NM MD, both required by SNPsplit (see below). After keeping only uniquely aligned reads and removing duplicates with Picard Tools (http://broadinstitute.github.io/picard/) — as suggested in Castel *et al.* (26) — reads were assigned to either the FVB or the PWD genome using SNPsplit (https://www.bioinformatics.babraham.ac.uk/projects/SNPsplit/). FVB reads and PWD reads were used for two separate instances of featureCounts (subreads v2.0.1) (13) to obtain gene counts, with parameters -s 0 -t 'exon' -g 'gene_id' -Q 0 --minOverlap 1 --fracOverlap 0 --fracOverlapFeature 0 -p -C. featureCounts output was then used in a custom Rmd for the analysis of ploidy, for which all gene counts for the FVB and PWD genome were summed for each embryo (excluding genes on sex chromosomes).

Additional details on statistical analyses

All statistical tests used are specified in the figure legends. The p-values are indicated in the figures, while adjusted p-values are indicated in figures only when <0.05 for single copy genes (except Btgh/dBtgh in *SI Appendix*, Fig. S2E because all the values are informative) or <0.1 for retrotransposons; higher values are considered not significant. For box plots, hinges correspond to first and third quartiles; median is shown inside; whiskers extend to the largest and smallest values no further than 1.5 * IQR from the hinge (IQR = inter-quartile range, or distance between the first and third quartiles); data beyond the end of the whiskers are plotted individually as single dots. Whenever MA-plots are shown, the $\log_2$ fold changes are shrunken using the 'ashr' method (18).

Supplemental References

1. J. Wu, *et al.*, The landscape of accessible chromatin in mammalian preimplantation embryos. *Nature* **534**, 652–657 (2016).
2. Y. Zhang, et al., Dynamic epigenomic landscapes during early lineage specification in mouse embryos. *Nature Genetics* **50**, 96–105 (2017).
3. C. C. Conine, F. Sun, L. Song, J. A. Rivera-Pérez, O. J. Rando, Small RNAs Gained during Epididymal Transit of Sperm Are Essential for Embryonic Development in Mice. *Developmental Cell* **46**, 470-480.e3 (2018).
4. A. Nakao, M. Yoshihama, N. Kenmochi, RPG: the Ribosomal Protein Gene database. *Nucleic Acids Res.* **32**, D168–D170 (2004).
5. M. Boulard, S. Rucli, J. R. Edwards, T. H. Bestor, Methylation-directed glycosylation of chromatin factors represses retrotransposon promoters. *Proc National Acad Sci* **117**, 14292–14298 (2020).
6. A. Eid, D. Rodriguez-Terrones, A. Burton, M. E. Torres-Padilla, SUV4-20 activity in the preimplantation mouse embryo controls timely replication. *Genes & Development* **30**, 2513–2526 (2016).
7. R. J. Dennis, *et al.*, Structure and mechanism of a bacterial β-glucosaminidase having O-GlcNAcase activity. *Nature Structural and Molecular Biology* **13**, 365–371 (2006).
8. S. Picelli, *et al.*, Full-length RNA-seq from single cells using Smart-seq2. *Nature Protocols* **9**, 171–181 (2014).
9. B. P. Hennig, *et al.*, Large-scale low-cost NGS library preparation using a robust Tn5 purification and tagmentation protocol. *G3: Genes, Genomes, Genetics* **8**, 79–89 (2018).
10. Y. Li, *et al.*, Click-iT ®; Plus OPP Alexa Fluor ®; Protein Synthesis Assay in Embryonic Cells. *BIO-Protoc.* **12**, e4441 (2022).
11. E. Afgan, *et al.*, The Galaxy platform for accessible, reproducible and collaborative biomedical analyses: 2018 update. *Nucleic Acids Research* **46**, W537–W544 (2018).
12. A. Dobin, *et al.*, STAR: ultrafast universal RNA-seq aligner. *Bioinformatics* **29**, 15–21 (2013).
13. Y. Liao, G. K. Smyth, W. Shi, featureCounts: an efficient general purpose program for assigning sequence reads to genomic features. Bioinformatics **30**, 923–930 (2014).
14. M. I. Love, W. Huber, S. Anders, Moderated estimation of fold change and dispersion for RNA-seq data with DESeq2. *Genome Biology* **15**, 550 (2014).
15. D. Risso, J. Ngai, T. P. Speed, S. Dudoit, Normalization of RNA-seq data using factor analysis of control genes or samples. *Nat Biotechnol* **32**, 896–902 (2014).
16. R. Patro, G. Duggal, M. I. Love, R. A. Irizarry, C. Kingsford, Salmon provides fast and bias-aware quantification of transcript expression. *Nat. Methods* **14**, 417–419 (2017).
17. C. Soneson, M. I. Love, M. D. Robinson, Differential analyses for RNA-seq: transcript-level estimates improve gene-level inferences. *F1000Research* **4**, 1521 (2016).
18. M. Stephens, False discovery rates: a new deal. *Biostatistics* **18**, 275–294 (2017).
19. D. Garrido-Martín, E. Palumbo, R. Guigó, A. Breschi, ggsashimi: Sashimi plot revised for browser- and annotation-independent splicing visualization. *PLoS Comput. Biol.* **14**, e1006360 (2018).
20. T. Wu, *et al.*, clusterProfiler 4.0: A universal enrichment tool for interpreting omics data. *Innovation* **2**, 100141 (2021).
21. G. Yu, *et al.*, GOSemSim: an R package for measuring semantic similarity among GO terms and gene products. *Bioinformatics* **26**, 976–978 (2010).
22. F. Mölder, *et al.*, Sustainable data analysis with Snakemake. *F1000Research 2021 10:33* **10**, 33 (2021).
23. A. Teissandier, N. Servant, E. Barillot, D. Bourc'His, Tools and best practices for retrotransposon

analysis using high-throughput sequencing data. *Mobile DNA* **10**, 52 (2019).
24. M. A. Bogue, *et al*., Mouse Phenome Database: towards a more FAIR-compliant and TRUST-worthy data repository and tool suite for phenotypes and genotypes. *Nucleic Acids Res* 51, D1067–D1074 (2022).
25. A. R. Quinlan, I. M. Hall, BEDTools: a flexible suite of utilities for comparing genomic features. *Bioinformatics* **26**, 841–842 (2010).
26. S. E. Castel, A. Levy-Moonshine, P. Mohammadi, E. Banks, T. Lappalainen, Tools and best practices for data processing in allelic expression analysis. *Genome Biology* **16** (2015).
